# A novel computational model of swine ventricular myocyte reveals new insights into disease mechanisms and therapeutic approaches in Timothy Syndrome

**DOI:** 10.1038/s41598-024-80726-2

**Published:** 2024-11-30

**Authors:** Alessandro Trancuccio, Carmen Tarifa, Rossana Bongianino, Silvia G. Priori, Demetrio J. Santiago

**Affiliations:** 1 Molecular Cardiology Unit, IRCCS Istituti Clinici Scientifici Maugeri, Pavia, Italy; 2 Department of Molecular Medicine, University of Pavia, Pavia, Italy; 3 Novel Arrhythmogenic Mechanisms Program, Centro Nacional de Investigaciones Cardiovasculares (CNIC), Madrid, Spain

**Keywords:** Mathematical Modeling, Cardiac Electrophysiology, Long QT Syndrome, CACNA1C, Swine Ventricular Myocyte, Computational models, Arrhythmias

## Abstract

Timothy syndrome type 1 (TS1), a malignant variant of Long QT Syndrome, is caused by L-type Ca2+ Channel (LTCC) inactivation defects secondary to the p.Gly406Arg mutation in the *CACNA1C* gene. Leveraging on the experimental *in vitro* data from our TS1 knock-in swine model and their wild-type (WT) littermates, we first developed a mathematical model of WT large white swine ventricular cardiomyocyte electrophysiology that reproduces a wide range of experimental data, including ionic current properties, action potential (AP) dynamics, and $\hbox {Ca}^{2+}$ handling. A sensitivity analysis tested robustness and facilitated comparison with the parent ORd human model. Introducing 22% of TS1-mutated LTCCs, the model faithfully reproduced key disease features, including marked AP prolongation, steeper rate-dependent adaptation of AP duration, $\hbox {Ca}^{2+}$ overload, and CaMKII-mediated decreased upstroke velocity. Translational relevance of the TS1 model was investigated by: dissecting the roles of primary and secondary contributors to TS1 phenotype; demonstrating the arrhythmogenic potential of TS1 vs. WT cells; and evaluating the model’s capability to identify novel pharmacological targets which could modulate the cellular phenotype. In conclusion, we developed a mathematical large white swine ventricular myocyte model, demonstrating its utility in exploring arrhythmogenic mechanisms and therapeutic interventions in cardiac diseases, such as TS1.

## Introduction

Timothy syndrome, known as Long QT syndrome type 8, a multiorgan and highly malignant variant of Long QT Syndrome (LQTS), is characterized by extreme QT interval prolongation and high incidence of life-threatening arrhythmias^1^. About 70% of affected individuals harbor the p.Gly406Arg *gain-of-function* mutation in the *CACNA1C *gene (Timothy Syndrome Type 1, TS1), which has been shown to impair the voltage-dependent inactivation (VDI) of the LTCC and thus prolong action potentials (APs)^1^. However, evidence from later works indicates that the ontology of arrhythmias in TS1 is more nuanced than previously anticipated. In addition to the primary contribution of mutated Ca_V_1.2s to phenotype^1^, secondary contributions have been identified. Specifically, cellular $\hbox {Ca}^{2+}$ overload activates CaMKII, which in turn causes excess Ca_V_1.2 phosphorylation^2^, increased late Na^+^ current (I_NaL_; e.g^3^.) and reduced peak I_Na_, the latter having been shown to be responsible of arrhythmogenic slowing of cardiac conduction *in vivo*^4^. Moreover, AP prolongation is thought to be aggravated by late-systolic $\hbox {Ca}^{2+}$ripples^4^, and a chronic I_Ks _reduction, resulting in further reduction of the repolarization reserve^4,5^. In summary, TS1 lethality would appear to stem from an overall remodelling of the normal architecture of the ionic fluxes that configure the AP, either as a primary effect of the mutation or as a result of secondary mechanisms. Such increasing complexity raises questions regarding the relative contributions of primary and secondary mechanisms to the TS1 phenotype and their therapeutic potential. Our study aims to address these questions by mechanistically dissecting the known contributors to the TS1 phenotype, using an *in silico* approach which leverages on the large amount of *in vitro* data collected in our recent characterization of a novel *knock-in *swine model of TS1^4^. Remarkable similarities between humans and pigs in terms of AP duration, ion channel profile and intracellular $\hbox {Ca}^{2+}$-handling dynamics render a porcine TS1 model a more clinically translatable model than previously utilized disease models (e.g., guinea pig^2^). In the broader perspective of mounting interest in the use of porcine models for cardiovascular research^6,7^, best corroborated by the recent porcine-to-human cardiac xenotransplantation^8^, the scarcity of single-cell electrophysiological data for swine cardiomyocytes represents an unmet need. While research works conducted over the past three decades have yielded *in silico *models for different animal species, including guinea pigs^9–11^, rabbits^12–14^, dogs^15,16^, and humans^17–19^, only one swine model of ventricular cardiomyocyte has been developed hitherto^20^ (Supplementary Figure 1). In this context, our wild-type (WT) swine model, robustly validated against extensive *in vitro* data collected at our laboratory, will represent an useful tool for preclinical research and might be utilized as a framework to explore arrhythmogenic mechanisms and pharmacological interventions in cardiovascular diseases.

## Methods

### Model development strategy

We developed a novel WT swine ventricular myocyte model, adapting it from the ORd human ventricular myocyte model^17^, with several modifications. Our aim was to reproduce the p.Gly406Arg mutation in the *CACNA1C *gene, which impairs the VDI of the LTCC. To achieve this, we replaced the ORd model’s existing formulation of the LTCC, based on the Hodgkin-Huxley (HH) formalism, with the Markov model by Bartolucci et al.^21^. This model represents VDI and $\hbox {Ca}^{2+}$-Dependent Inactivation (CDI) as two separate loops, each with four states: closed (C), open (O), and two inactivated states (I1 and I2). Additionally, we incorporated several modifications based on experimental evidence in swine^4^. Specifically, we made Na_v_1.5 conductance CaMKII-dependent^4^. Since there is no evidence of I_to1_ in the swine, but I_to2 _is significantly present^22^, we removed I_to_ and integrated the I_to2 _formulation from Hund-Rudy^16^. Our model’s use of a Markovian I_Ca_ formulation validated against Ba^2+^ and $\hbox {Ca}^{2+}$ currents, combined with a single $\hbox {Ca}^{2+}$ release compartment and CaMKII-mediated modulation of Na^+^ currents (both fast I_Na_ and I_NaL_), distinguishes it from the model by Gaur et al.^20^. The final myocyte model comprises 61 ordinary differential equations describing the rate of change of membrane potential, ion channel gating kinetics, intracellular Na^+^, K^+^, and $\hbox {Ca}^{2+}$ concentrations, and intracellular $\hbox {Ca}^{2+}$ cycling dynamics. All model equations are detailed in the Appendix Section within the Supplementary Material.

### Fitting of gating parameters of ionic currents

Model parametrization was based on swine data from our laboratory, including voltage-clamp data for I_Na_, I_NaL_, I_Ba_, I_Ca_, I_K1_, I_Kr_, and I_Ks_, as well as current-clamp data for APs and $\hbox {Ca}^{2+}$transients (CaTs)^4^. Additional experimental data, such as recordings of I_to2_ and APD90 restitution at 1 Hz, were collected specifically for this study (Experimental Methods Section). Parameter values for ion current gates (I_Na_, I_NaL_, I_Ba_, I_Ca_, I_Kr_, I_Ks_, I_K1_, I_to2_) were obtained by fitting experimentally measured I-V relationships, activation and availability curves, Fractional Release Current (FRC), and recoveries from inactivation. Automated fitting was conducted using the Nelder-Mead algorithm^23^. The cost function was calculated as the sum of squared differences between simulated and experimental data, each weighted according to specific experimental conditions (e.g. number of sweeps):

$$\begin{aligned} \text {Error} = \text {weight}_i \cdot \sum (Y_{i,\text {sim}} - Y_{i,\text {exp}})^2 \end{aligned}$$

Parameters before and after fitting are summarized in Supplementary Tables 1–7. We retained the parameter values for $\hbox {Ca}^{2+}$buffering (except for Km of TRPN, modified from^24^), NCX, the sarcolemmal $\hbox {Ca}^{2+}$ pump, and background $\hbox {Ca}^{2+}$, Na^+^, and K^+^ currents from the ORd model. For the sarcolemmal Na^+^/K^+^ pump, we adjusted MgATP levels to match our experimental conditions. We also modified ionic conditions, including intracellular and extracellular Cl^-^ concentrations (not included in ORd), to align with our experimental values.

### Cell capacitance and dimensions

We assumed cylindrical cell shape with a length of 0.0105 cm and a diameter of 0.0012 cm, based on our experimental data. The dimensions of the intracellular compartments, expressed as a percentage of cell volume, were adopted from ORd model^17^.

### Membrane currents

Herein, a brief description of Na^+^, K^+^ (I_Kr_, I_Ks_, I_K1_), Cl^-^ (I_to2_) and L-type (I_Ba_, I_Ca_) currents is provided.

***Na***^+^*** Current:*** The fast Na^+^ current (I_Na_) was formulated using the HH formalism as in ORd^17^. We modified the gating parameters to fit experimental voltage-clamp data from swine ventricular myocytes^4^. Experimental protocols included: i) voltage-dependence of activation; ii) voltage-dependence of inactivation; iii) recovery from fast inactivation; iv) normalized peak I-V curve. Since I_Na _recordings were performed at 22°C, a temperature correction was applied to simulate proper kinetic behavior at 37°C, as per the methodology in ORd model^17^. The effect of temperature on steady-state gating was assumed to shift by +4.3 mV and +4.7 mV for V_1/2 _of activation and inactivation, respectively, with a temperature change from 23°C to 33°C^17^. Time constants were adjusted to 37°C using a Q_10 _value of 2, as in ORd^17^. Prior to final model optimization (see methods below), manual adjustment of I_Na_ conductance (G_Na_) was achieved to match the experimentally measured maximum upstroke velocity (max. dV/dt) at three frequencies (0.5, 1, and 2 Hz). CaMKII dependency of G_Na_ was further added, to match experimental evidence of CaMKII-mediated reduction in peak I_Na_ in TS1 (see TS1 model below). Furthermore, the I_NaL_ conductance and the time constant of the *hL *inactivation gate were adjusted to match the end-pulse experimental current density (−0.12 pA/pF^4^) and kinetics (measured time constant of inactivation of 175 ms) of TTX-sensitive current upon a depolarizing pulse from −120 mV to −30mV recorded at 22ºC^4^. Similarly to fast I_Na_, dependence of G_NaL_ on CaMKII levels was added to match experimental evidence of CaMKII-mediated increased in I_NaL_ current in TS1^4^ (see TS1 model below).

***K***^+^*** Currents***: The formulations of I_Kr_, I_Ks_, and I_K1_were based on the HH formalism. Model equations are detailed in the Appendix Section within the Supplementary Material. Gating parameters and conductances were modified to fit experimentally measured peak I-V curves^4^.

**Calcium-activated Chloride Current (I**_to2_**)**: In humans, the transient outward current (I_to_) primarily consists of the I_to1_ component, driven by K^+^ currents. However, in pigs, I_to_ is mainly attributed to the $\hbox {Ca}^{2+}$-activated chloride current, known as I_to2_, as elucidated by Rong Li et al.^22^. To model this difference, we adapted equations from the Hund and Rudy model^16^, fine-tuning the parameters to align with experimental data. Methods for I_to2_ recordings are detailed in the Experimental Methods Section.

***L-type Currents:*** To separately assess VDI and CDI, we leveraged our recently published experimental recordings using Ba^2+^ as the charge carrier (allowing only VDI) and $\hbox {Ca}^{2+}$as the charge carrier (allowing both CDI and VDI)^4^. The equation parameters were adjusted to fit the experimental data, including: 1) voltage-dependence of activation, 2) voltage-dependence of inactivation, 3) recovery from inactivation, 4) peak I-V curve, 5) Fractional Remaining Current (FRC). Following previously published methodology^17^, FRC was used to quantify the voltage and time dependencies of inactivation. Specifically, at time t (after the peak) and voltage V_m_, FRC is expressed as:

$$\begin{aligned} \text {FRC}(t, V_m) = \frac{I(t, V_m)}{I_{\text {peak}}(V_m)} \end{aligned}$$

### Automatic optimization procedure

After the aforementioned fitting of ionic currents gating parameters, we focused on the behavior of current-clamped cells. In particular, and given the biological variability of ionic conductances in the heart, similarly to previous studies in different models^21,25^, an automatic optimization was performed to fine-tune main ionic conductances, $\hbox {Ca}^{2+}$ release and $\hbox {Ca}^{2+}$ uptake parameters. The cost function of the optimization procedure was based on quantitative data on AP and CaT features at 0.5, 1 and 2 Hz pacing frequency, including: AP duration at 20, 50, and 90% repolarization (APD20, APD50, and APD90), max dV/dt, action potential amplitude (APA), resting membrane potential (RMP), CaT amplitude, CaT time to peak, CaT full duration at half magnitude (FDHM) and CaT time to 90% decay from peak. As an additional refinement constraint, we imposed for the model to reproduce the biological APD90 restitution behavior, for which we collected data at 1 Hz. After each simulation, the set of quantitative descriptors was extracted and compared with the experimental data. The search for the optimal solution was conducted using the Nelder-Mead simplex method. Initial and final parameters are reported in Supplementary Table 8.

### Sensitivity analysis

We performed a sensitivity analysis following the approach by Sobie et al.^26^, which has been re-adopted by others^25,27^. This analysis involved a randomization procedure designed to replicate the biological variability of ion channel conductances, $\hbox {Ca}^{2+}$ release, and $\hbox {Ca}^{2+}$ uptake fluxes (i.e., the model parameters, or “inputs”), which translates into biological variability of cellular action potentials (APs) and $\hbox {Ca}^{2+}$ transients (CaTs) (i.e., the model responses, or “outputs”). This analysis was performed at 1 Hz pacing. The randomization procedure involved the same parameters that underwent automatic optimization, for a total of 14 parameters. Parameters were randomized through scaling factors chosen from a log-normal distribution with a median value of one and a standard deviation of 0.2. This implies that each parameter was randomly varied between 0.5 and 1.5 times the values used in the model. The randomization was run for a population of 500 models, each of which was simulated according to its inputs, and the corresponding AP and CaT features were computed. To allow for inputs and outputs (which are expressed in different units) to be comparable, values in the input matrix $\textbf{X}$ and the output matrix $\textbf{Y}$ were converted into Z-scores – i.e., each column was mean-centered and normalized by its standard deviation. From this input matrix of parameters and the output matrix of responses, we derived a matrix $\textbf{B}$ that summarizes the relationship between model parameters and responses. Given matrices $\textbf{X}$ ($n \times p$) and $\textbf{Y}$ ($m \times n$), where $n$ is the number of simulations ($n=500$), $p$ is the number of parameters, and $m$ is the number of computed features, the sensitivity coefficients matrix $\textbf{B}$ ($p \times m$) was computed using the formula^26^:

$$\begin{aligned} \textbf{B} = (\textbf{X}^{T} \times \textbf{X})^{-1} \times \textbf{X}^{T} \times \textbf{Y} \end{aligned}$$

where $\textbf{X}^{T}$ indicates the transpose of $\textbf{X}$. We repeated the sensitivity analysis in the parent ORd subjected to our experimental conditions to identify and rank the most sensitive factors explaining each biological response in the human model compared to the swine model.

### Swine Timothy Syndrome model

The TS1-causative p.Gly406Arg mutation in *CACNA1C *was simulated by introducing a second population of mutated LTCCs into the aforementioned WT swine model. In particular, the population of mutated LTCCs experienced a slowing of the transition rates from O to I1 and from C to I2. Only those 2 parameters were varied in order to fit experimental data from Ba^2+^ current recordings (VDI only) and $\hbox {Ca}^{2+}$ current recordings (VDI+CDI) obtained from cardiomyocytes of G406R-mutant pigs. The proportion of mutated channels in the model was estimated to be 22%, based on experimental data (See Results). In addition to the effect of the mutation on the LTCC, we made appropriate modifications to account for important findings from our *in vitro *phenotypical characterization of the knock-in swine model of TS1^4^. These modifications included: 1) 40% reduction in I_Ks_ current density in TS1 compared to WT, in the absence of $\hbox {Ca}^{2+}$regulation^4^; 2) positive (+20.7 mV) shift and 5% reduced conductance of I_to2_ in TS1 (new experimental data); 3) a CaMKII-dependency of G_Na _that is shared with the WT model, capable of reproducing the experimentally measured reductions in AP’s upstroke velocity in current-clamped TS1 vs. WT cells (0.5, 1 and 2 Hz at physiological temperature)^4^; 4) a CaMKII-dependency of G_NaL_ that is shared with the WT model, capable of reproducing a two-fold increase in I_NaL_in TS1 vs. WT myocytes^4^. For the TS1 model validation, we required of the model to accurately reproduce not only the significant AP prolongation but also capture other relevant pathological features, including: increased steepness of APD rate adaptation, CaMKII-mediated rate-dependent decrease in AP upstroke velocity, intracellular $\hbox {Ca}^{2+}$ overload, increased amplitude and altered kinetics of $\hbox {Ca}^{2+}$ transients, including increased duration, and capacity of producing EADs by well-known mechanisms (see Results). We also performed a sensitivity analysis following the same methodology used for the WT model.

### Simulations concerning EADs, gene therapy and pharmacological therapies

EAD-related simulations were performed by variations in the percentage of mutated Ca_v_1.2s, by slowing the time constant of hL gate, by left-shifting the voltage dependency of LTCC activation and/or by the mathematical imposition of cytosolic $\hbox {Ca}^{2+}$ripples/waves. This latter method is similar to the one used by the Weiss laboratory^28–30^, in which free diastolic [$\hbox {Ca}^{2+}$] is forced to follow a gaussian curve with known latency, width and magnitude, thus mathematically imposing diastolic $\hbox {Ca}^{2+}$ waves of known characteristics *in silico*. In our case, we simulated late-systolic $\hbox {Ca}^{2+}$ ripples by conferring a time-dependency with oscillatory behavior to the release parameter *bt*, starting after a latency of* t*_*Jlate* ms after cell stimulation. In that way, the imposition is on release itself, rather than systolic free $\hbox {Ca}^{2+}$. The following procedure was used:
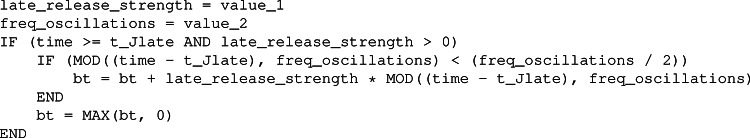


With this procedure, late-systolic releases of different magnitude, oscillatory frequency and timing could be imposed on the TS1 myocyte, creating a wide range of effects either in isolation or specially when combined with other EAD-generating mechanisms: from no EADs, to isolated EADs at different timepoints in the action potential, multi-oscillating EADs, and large APD alternations accompanied by EADs.

Gene therapy was simulated by multiplying the respective $\hbox {Ca}^{2+}$ currents of WT and TS1 Ca_v_1.2 subpopulations (in the absence of gene therapy) by the fraction of active channels in each subpopulation:

$$\begin{aligned} & \text {active}\_\text {WT}\_\text {LTCC} = 1 - \text {Desired}\_\text {Silencing}\_\text {WT} \times \text {gene}\_\text {therapy} \\ & \text {active}\_\text {TS1}\_\text {LTCC} = 1 - \text {Desired}\_\text {Silencing}\_\text {TS1} \times \text {gene}\_\text {therapy} \end{aligned}$$

where “gene_therapy” could take the values of zero (no silencing) or one (therapy applied). Given that there is no mechanistic explanation for the remodeling of I_to2_ and I_Ks_ in TS1, we simulated these phenotypes as simple linear dependencies on the %TS1 Ca_v_1.2s. When treated with gene therapy, the phenotypes further depended on the active TS1 channels. G_Ks_ serves here as example of the equations used:

$$\begin{aligned}G_{Ks,TS1}=G_{Ks,WT} \times \left(1-0.4 \times active\_TS1\_LTCC \times \frac {fraction\_TS1\_channels}{0.22}\right) \end{aligned}$$

where G_Ks _is the WT conductance for I_Ks_, 0.4 refers to the experimentally observed fractional change in conductance in TS1, and 0.22 is the experimentally observed fraction of mutated Ca_v_1.2s in TS1 cells. The effects of verapamil were simulated as in^31^, by scaling P_Ca_ by 0.64, G_Kr_ by 0.55, and G_Na_ by 0.998 (i.e. LTCC block with partial selectivity for the swine ERG channel, plus a marginal block of the Na_v_1.5 channel). The *in silico *effects of ICA-105574 on TS1 cells were simulated by first fitting our laboratory’s voltage-clamp results on expressed human ERG channels^32^, and then applying similar changes to the treated cell. In particular, we introduced a +42 mV shift in the inactivation gate, a −11 mV shift in activation, and a 2.277-fold increase in G_Kr_. Treatment with mexiletine and ranolazine was based on patch-clamp data at physiological temperature from Crumb *et al. *^33^. Specifically, we modeled the effects of 10 $\mu$M mexiletine as inducing a 52% block of $\hbox {I}_{NaL}$*, *along with 9% $\hbox {I}_{Kr}$, 20% $\hbox {I}_{Ca}$, and 6% $\hbox {I}_{Na}$ block. Similarly, 6.9 $\mu$M ranolazine was modeled to produce a 48% block of $\hbox {I}_{NaL}$, 54% block of $\hbox {I}_{Kr}$, and 6% block of $\hbox {I}_{Na}$.

### Hardware and software

Simulations were executed using MATLAB R2024a (Mathworks Inc., Natick, MA, United States) on a Windows 11 Pro (version 22H2) Lenovo computer (Lenovo Group Limited, Quarry Bay, Hong Kong). Numerical integration was performed by ode15s, a variable order solver based on numerical differentiation formulas, provided by MatLab (The Mathworks, Inc.). Simulations were run for 800 seconds, to ensure that steady-state was reached. During fits of voltage-clamp data, sweeps started from steady-state at the holding potential. For the ode15s, options were set to: RelTol 1e-10, AbsTol 1e-11, MaxStep 1. Automatic optimization, feature extraction and sensitivity analysis were performed by custom code in MatLab R2024a. MatLab code of the novel swine model is available at the following link: https://shorturl.at/tQiRN.

### Experimental methods

***Ethical Considerations:*** All experiments involving animals were performed in accordance with relevant guidelines and regulations after authorization by relevant authorities. Specifically, all animal protocols were approved by the Centro Nacional de Investigaciones Cardiovasculares (CNIC) in-house ethical committee, the Universidad Autónoma de Madrid and the Comunidad de Madrid (PROEX 152.7/22) and conform to European Union Directive 2010/63/EU. All authors complied with the guidelines for Animal Research: Reporting of In Vivo Experiments (ARRIVE).

***Ventricular myocytes isolation:*** we obtained ventricular myocytes from Large-White pigs of both sexes aged 4–6 weeks (weight range, 7–10 kg), using a Langendorff heart perfusion system and following exactly the same methodology and anesthesia procedures described in Porta-Sanchez et al.^4^.

***Single-cell electrophysiology:*** data were collected using an Axopatch 200B amplifier and pCLAMP software 10.4. Digitization was accomplished with a Digidata 1550B (Molecular Devices). Data were sampled at 10 kHz and filtered at 2 kHz.

***Action potential restitution at 1 Hz:*** WT swine myocytes were current-clamped at 36°C, using 1.5–4.5 M$\Omega$ pipettes. The external solution contained (in mM): 140 NaCl, 4 KCl, 10 HEPES, 5 glucose, 1 MgCl_2_, and 1.8 CaCl_2_; with the pH adjusted to 7.4 using NaOH. The internal solution contained (in mM): 120 K-Aspartate, 20 KCl, 4 Na_2_ATP, 0.4 GTP, 10 HEPES, 10 glucose, and 4.4 MgCl_2_ (free Mg^2+^ calculated to be 1 mM using MaxChelator software https://somapp.ucdmc.ucdavis.edu/pharmacology/bers/maxchelator/webmaxc/webmaxcS.htm), with pH adjusted to 7.2 using KOH. APs were elicited by 3 ms stimulations at injected currents of magnitude 20–25% above the AP threshold. Restitution curves (n = 4) were built by first pacing cells at 1 Hz for at least 60 beats. Then, a S1-S2 protocol with variable interpulse interval ( $\Delta$t = 10 ms) was performed. For each sweep, a (S1) train of 21 APs was acquired at 1 Hz and this was followed by the second stimulation (S2) at a variable diastolic interval, defined for analysis purposes as the time span from the APD90 of the 21^st^AP in the sweep. The liquid junction potential (−17.815mV^4^) was corrected prior to analysis. AP restitution data were analyzed using Iterative Data Language (version 8.1, Harris Geospatial).

***I***_to2 _***current recordings:*** I_to2_ was recorded using the whole-cell ruptured voltage-clamp technique (n = 2 WT vs. 3 TS1 animals, respectively 11 vs. 17 cells) at 36°C, utilizing 1–3 M$\Omega$ pipettes and the internal/external solutions used for AP restitution (above). I_to2_ currents were elicited after capacitance and series resistance compensation (70–80%, lag 20 $\mu$s) from a holding potential of −50 mV. A 10 ms pre-pulse to 20 mV was followed by 200 ms test pulses ranging from −40 to 70 mV. Ionic currents are presented in pA/pF. The $\hbox {Ca}^{2+}$ dependency of I_to2_ was confirmed by the use of 100 $\mu$M CdCl_2_^4^. I-V curves for I_to2_ were analyzed using Clampfit 10.6.0.13. In the I-V analysis, baseline offsets were adjusted before analysis, and corrections of leak were applied under the assumption of linearity at all test voltages.

***Splicing analysis of mutually exclusive exons 8/8A of the CACNA1C gene:*** to determine the splicing profile of the *CACNA1C *gene in TS1 animals, RNA was extracted from left ventricular biopsies (n=5 TS1 vs. n=5 WT pigs of both sexes), and retro-transcribed. Fragments from exon 7 to exon 10 were then amplified and cloned into pCR 4-TOPO TA vector (Thermo Fisher Scientific). PCR clones were transformed into One Shot TOP10 *E. coli* cells (Invitrogen). Screening and genotyping of positive colonies was performed by PCR amplification of the insert and sequencing. Data are reported as proportions.

***Statistics: ***sample sizes were based on similar experiments we conducted in our prior swine study^4^. No randomization or blinding was applied. No data were excluded during the analysis. Data on ionic currents are presented as mean±s.e.m., per convention and compared using repeated-measures two-way ANOVA with Sidak post-tests. Two-tailed *P* values were calculated with the statistical significance threshold set at *P*<0.05. Data were analyzed using GraphPad Prism version 8 (GraphPad Software).

## Results

### Development and validation of a novel wild-type swine ventricular cell model

**Fig. 1:**
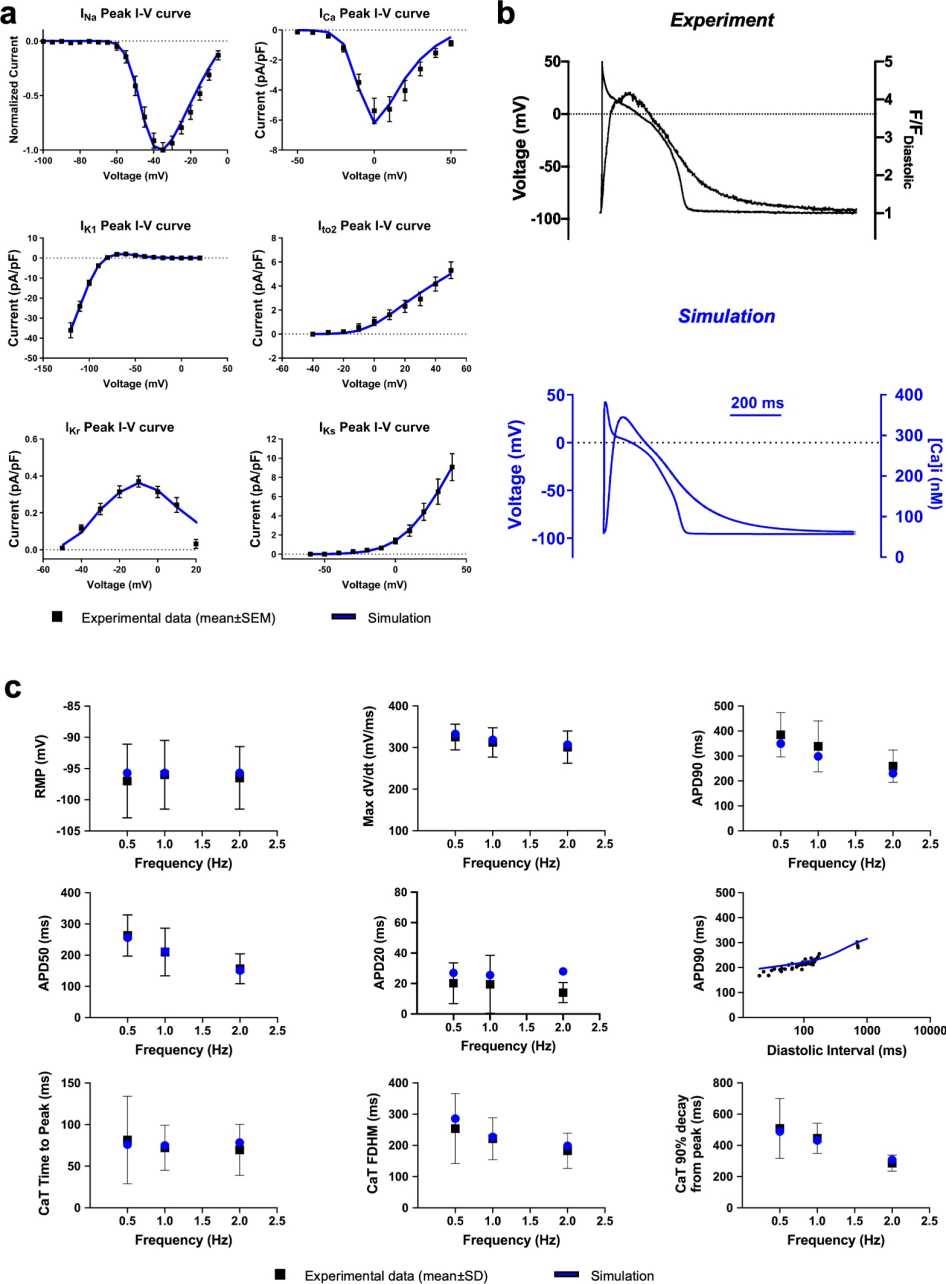
Development and validation of a novel swine model of ventricular action potential. (**A**) Comparison of the experimental (black, mean±SEM) and fitted (blue) IV curves for I_Na_, I_Ca_, I_K1_, I_to2_, I_Kr_ and I_Ks_. I_Na_ is presented as normalized current because it was recorded at room temperature, differently from the other currents which were recorded at physiological temperature. Therefore, the I_Na_ gating variables had to be temperature-corrected (see *Methods*). (**B**) Comparison of simulated (blue) and experimentally measured (black) action potentials (APs) and $\hbox {Ca}^{2+}$ transients (CaTs) during steady-state pacing at 1 Hz. (**C**) Biomarkers computed on simulated spontaneous APs and CaTs (blue) and their comparison with the experimental values (black, mean±SD). Experimental data derived from Porta-Sanchez et al.^4^, with the exception of novel I_to2_ current recordings.

**Fig. 2:**
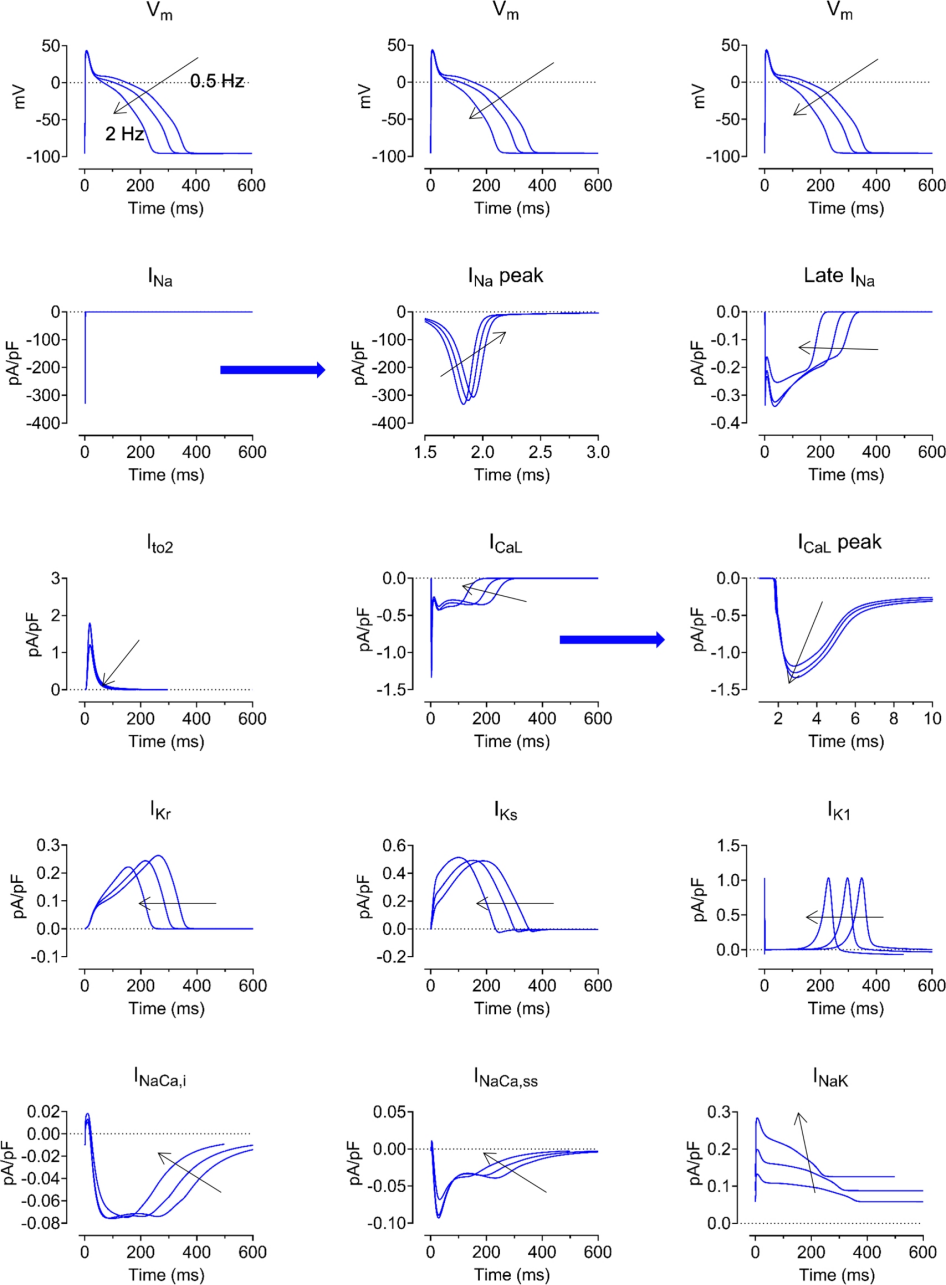
Rate dependence of currents at steady state in the novel swine model of ventricular action potential. Rate dependence of currents at steady state. Black arrows indicate cycle length decrease (rate increase). *Top Row:* Simulated APs, repeated in each column for timing purposes. *Lower Rows* (left to right, top to bottom): I_Na_, peak I_Na_ detailed time course, late I_Na_, I_to2_, I_CaL_, I_CaL_ increasing peaks with increasing pacing rate, I_Kr_, I_Ks_, I_K1_, I_NaCa,i_ (cytosol), I_NaCa,ss_ (subsarcolemmal), and I_NaK_.

Fitting ion currents gating parameters (I_Na_, I_NaL_, I_Ba_, I_Ca_, I_Kr_, I_Ks_, I_K1_ and I_to2_) involved I-V relationships, activation curves, availability curves, FRC and recoveries from inactivation (Figure 1A, Supplementary Figures 2-3). Subsequently, automated parameter optimization followed previously published methods^21,25^, with a cost function ensuring simulation results matched quantitative experimental data on AP and CaT features at 0.5, 1, and 2 Hz (Figure 1B-C). Simulated AP waveforms closely matched experimental traces across all frequencies tested, recapitulating morphological characteristics, such as the notch dome (Figure 1B). Quantitative AP parameters were within experimental mean±SD ranges (Figure 1C), and steady-state APD90 rate dependence and AP restitution curves also aligned well with experimental findings. Furthermore, simulated CaTs qualitatively and quantitatively reproduced experimental data across pacing frequencies (Figure 1B-C). Figure 2 illustrates the rate dependence of the major ionic currents responsible for AP shaping.

**Fig. 3:**
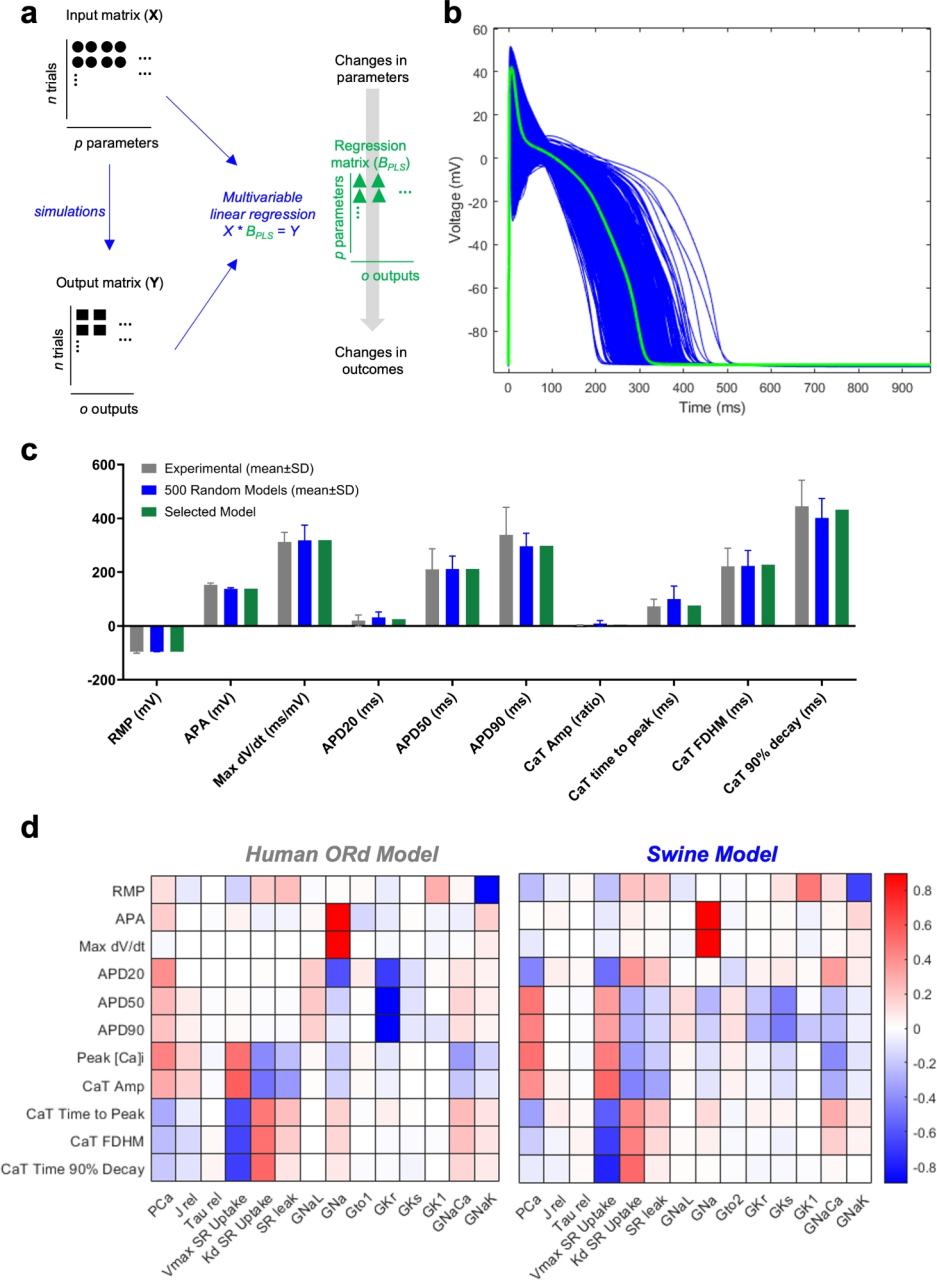
Sensitivity Analysis conducted in the new swine model and comparison with the human ORd parent model. (**A**) Schematic illustration of the approach adopted to perform parameter sensitivity analysis. The input matrix **X** contains randomly generated scaling factors (represented by circles) used to perturb the values of selected parameters in the model. For each simulation, AP and CaT features (squares) are estimated at steady state and their values collected in the matrix **Y**. Multivariable regression analysis between the input matrix **X** and the output matrix **Y** is performed to assess the sensitivity of AP and CaT features to changes in model parameters. The result of this process is the regression matrix **B**, which coefficients (triangles) quantify the sensitivity of model features to parameter perturbations. (**B**) APs at the steady state at 1 Hz for the 500 randomly generated models (blue), along with the final selected model (green). (**C**) Biomarkers computed on simulated APs and CaTs (blue) for the 500 randomly generated models (blue, mean±SD) and their comparison with experimental values (gray, mean±SD) and with the final selected model (green). (**D**) Colour coded map of sensitivity matrix **B **obtained from the human ORd parent model (*left*) and the novel swine model (*right*). Columns show how a specific model parameter affects AP and CaT features; rows show how each feature is affected by different parameters. Red, blue and white pixels represent positive, negative and no substantial correlation, respectively, between parameters and features.

Subsequently, we conducted a sensitivity analysis following the method by Sobie et al.^25–27^. This involved generating 500 models to account for biological variability by varying ion channel conductances, $\hbox {Ca}^{2+}$ release, and $\hbox {Ca}^{2+}$ uptake fluxes. Regression coefficients were derived to quantify the influence of each parameter (e.g., G_Na_) on specific cellular responses (e.g., max dV/dt) (Figure 3A). All 500 simulations were valid, evidenced by absence of APD/CaT alternans and repolarization failures (Figure 3B). Parameters obtained through automated optimization closely matched the average of the randomly generated models (Figures 3C). Figure 3D illustrates the sensitivity matrix **B**, where each value represents how changes in a parameter (displayed at the bottom) affect a specific feature (displayed on the left). The maintenance of RMP primarily depended on I_K1_ and I_NaK_ conductances, akin to the parent ORd model. Fast I_Na_ predominantly influenced AP amplitude and maximal upstroke velocity. Variations in I_Ca_, I_NaL_, I_Ks_, I_Kr_, and I_K1_ conductances significantly affected AP duration (APD20, APD50, and APD90). Notably, unlike the ORd model, where I_Kr_ is the major determinant of AP repolarization, in our swine model we observed that repolarization reserve relies more heavily on I_Ks_. Furthermore, our analysis highlighted that the maximal activity of the SERCA pump, its dissociation constant and the NCX conductance have a stronger impact on the kinetic features of the AP than in the ORd model.

### Development and validation of the TS1 swine ventricular cell model

**Fig. 4:**
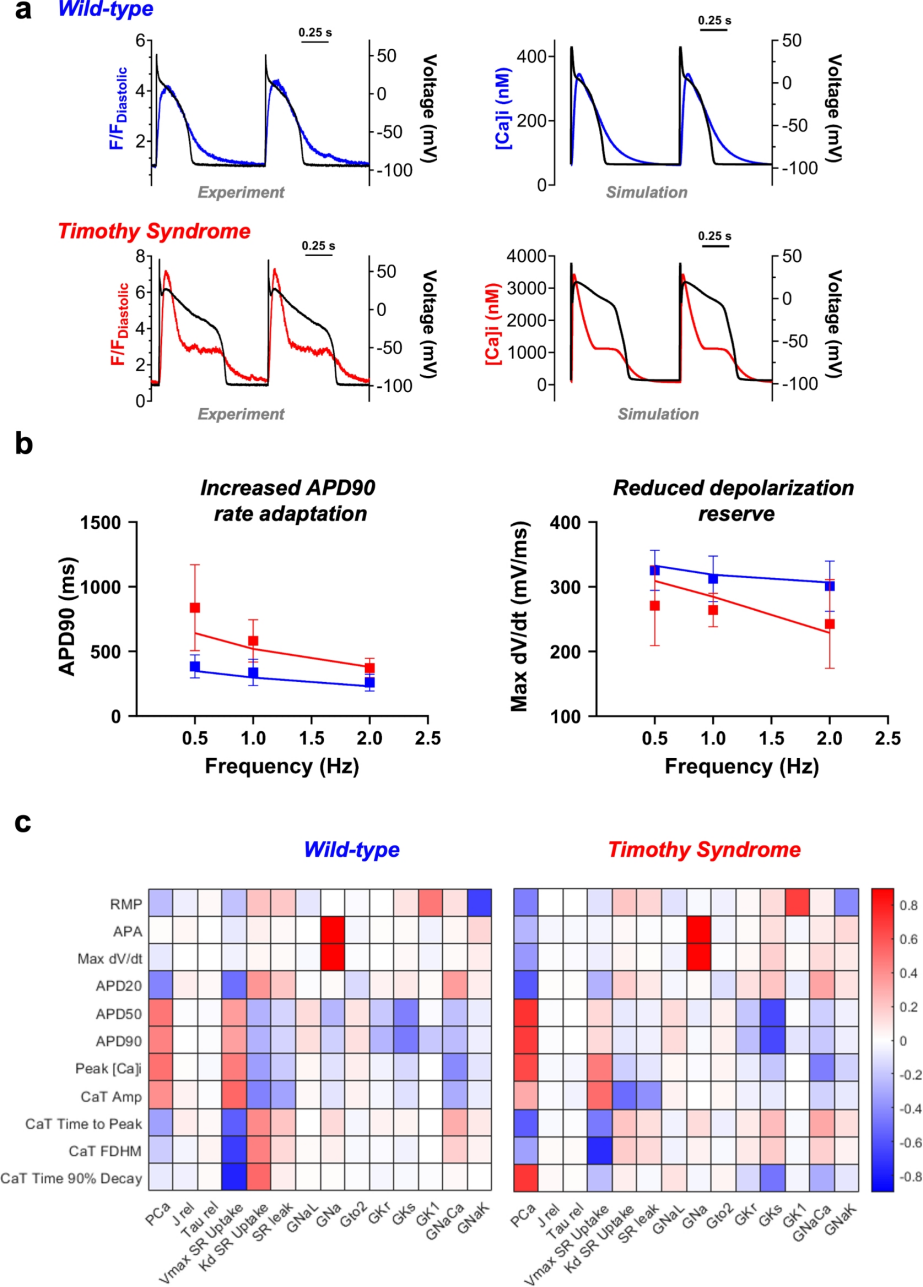
TS1 swine model of ventricular action potential and $\hbox {Ca}^{2+}$ handling. (**A**) Comparison between experimental (*left*) and simulated (*right*) WT and TS1 APs and CaTs at 1 Hz pacing frequency. Membrane voltage is plotted on the left axis, while [$\hbox {Ca}^{2+}$]i (or fluorescence for experimental data) is plotted on the right axis. (**B**) Comparison between experimental (squares, mean±SD) and simulated (line) APD90 rate adaptation (*left*) and maximum dV/dt (*right*) for WT (blue) and TS1 (red). (**C**) Sensitivity analysis (as in Figure 3). Colour-coded sensitivity matrix **B **obtained from the WT swine model (left) and the TS1 swine model (right). Experimental data from Porta-Sanchez et al.^4^.

Following the development and validation of the WT swine ventricular myocyte model, we formulated and validated the TS1 swine ventricular cardiomyocyte model. The TS1-causative mutation was simulated by introducing a second population of mutant LTCCs, comprising 22% of total LTCCs, based on experimental data specifically collected for this study (see below). LTCC modifications were initially validated with Ba^2+^ current recordings (VDI only) and subsequently with $\hbox {Ca}^{2+}$ current recordings (VDI+CDI) (Supplementary Figure 4). Also, we verified that simulating the voltage-dependence of Ba^2+^ current inactivation with 100% TS1-LTCCs qualitatively reproduced the experimental data obtained in CHO cells by Splawski et al.^1^ (Supplementary Figure 5 and refer to Figure 5-I in Splawski et al.^1^ for comparison). Figure 4 compares WT and TS1 AP and CaT traces at 1 Hz (Panel A) and pooled data at 3 pacing frequencies (Panel B), showing experimental results (top) *vs. *simulations (bottom). To align with phenotypic features observed in the *knock-in *swine model of TS1^4^, we implemented the following modifications: 1) CaMKII-mediated reduction in peak I_Na_; 2) CaMKII-dependent increase in late I_Na_; 3) 40% reduction in I_Ks_ current density, in the absence of $\hbox {Ca}^{2+}$regulation^4^; and 4) 5% decrease in conductance and +20.7 mV shift in I-V relationship of I_to2_ in TS1 (Supplementary Figure 6). Importantly, the model predicts significant AP prolongation in TS1 and recapitulates other pathological features: 1) increased APD90 rate adaptation; 2) reduced depolarization reserve, particularly with higher stimulation frequency; and 3) intracellular $\hbox {Ca}^{2+}$ overload, leading to prolonged CaT duration and increased amplitude (Figure 4). The model also replicates the distinctive CaT morphology in TS1, characterized by an initially higher and narrower phase followed by a sustained late-plateau with elevated $\hbox {Ca}^{2+}$ concentration (Figure 4). These features validate the model’s ability to replicate the complex TS1 cellular phenotype beyond AP prolongation. As above, we conducted the sensitivity analysis in the TS1 model, following the same methods and criteria than in the WT model. Out of 500 randomly generated models, 444 (89%) simulations were considered valid (i.e. absence of APD/CaT alternans and/or repolarization failures), in agreement with the notion that TS1 cells are more sensitive to abnormalities in repolarization. In the regression matrix (Figure 4C), we noted a similar overall pattern to the WT model, except for a greater impact of LTCC permeability variations on AP and CaT features, consistent with TS1 pathophysiology. Notably, the TS1 myocyte model exhibited a slight negative correlation between P_Ca_and max dV/dt, a characteristic absent in the WT model, aligning with the CaMKII-dependent conduction delay experimentally observed in TS1^4^. The time courses of major ionic currents influencing AP characteristics in WT versus TS1 at 1 Hz are depicted in Supplementary Figure 7.

### Dissecting the role of primary vs. secondary contributors to the TS1 cellular phenotype

Besides the primary role of mutated Ca_V_1.2 channels^1^, other secondary mechanisms contribute to the TS1 phenotype, in particular increased CaMKII activation due to $\hbox {Ca}^{2+}$ overload and remodeling of repolarizing currents. However, the relative impact and therapeutic potential of these secondary mechanisms remain unclear. Here, we used our *in silico* swine TS1 ventricular myocyte model to analyze the roles of primary *vs.* secondary contributors to the TS1 phenotype and their therapeutic potential.

#### Primary contribution of mutated Ca_V_1.2 channels

**Fig. 5:**
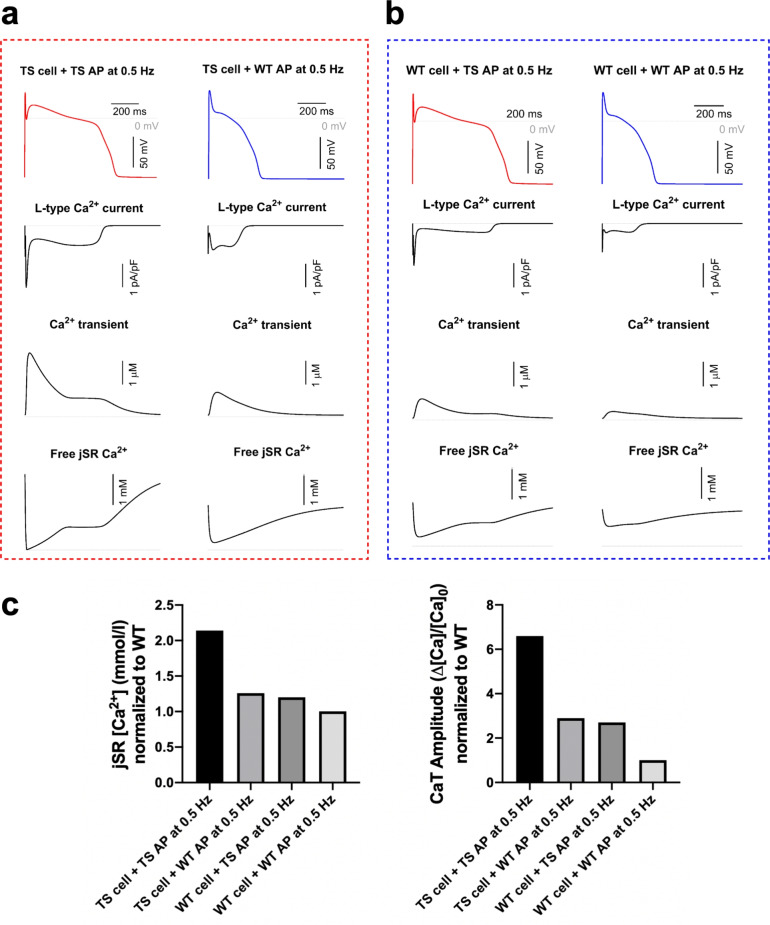
Simulated action potential-clamp in WT and TS1 ventricular models. (**A**) Simulations of TS1 cell subjected to TS1 AP at 0.5 Hz (left, i.e. “original TS1 cell”) and TS1 cell subjected to WT AP at 0.5 Hz (right). (**B**) Simulations of WT cell subjected to TS1 AP at 0.5 Hz (left) and WT cell subjected to WT AP at 0.5 Hz (i.e. “original WT cell”). From top to bottom: APs, LTCC Current, CaT and jSR [$\hbox {Ca}^{2+}$]. (C) Quantification of jSR $\hbox {Ca}^{2+}$ and CaT amplitude in the different situations. Absolute WT values: jSR $\hbox {Ca}^{2+}$ = 1.15 mM; CaT amplitude = 4.2.

To investigate the primary contribution of mutated Ca_V_1.2 channels, we assessed whether TS1 mutation’s effects were solely due to AP prolongation. Using a method similar to the experimental one in a TS1 mouse model by Drum et al.^34^, WT and TS1 myocytes were depolarized with either a WT or TS1 AP through simulated AP-clamp technique. As shown in Figure 5, when comparing TS1 cells under the TS1 AP to WT cells under the TS1 AP, intracellular $\hbox {Ca}^{2+}$ abnormalities were more severe when AP prolongation resulted from the TS1 mutation. Comparing WT cells under the TS1 AP to TS1 cells under the WT AP showed similar SR $\hbox {Ca}^{2+}$overload and CaT magnitudes, both differing from WT cells under WT AP. This suggests that restoring WT AP duration in TS1 cells does not fully normalize their phenotype. Also, the TS1 AP induced a significant late-phase plateau in the CaT in both phenotypes, consistent with data in failing myocytes where AP shape determines late-systolic release^35^. Our findings show that $\hbox {Ca}^{2+}$ abnormalities in TS1 arise not just from AP prolongation, but specifically from the LTCC abnormality causing it, distinguishing TS1 from other forms of LQTS.

#### Targeting the primary mechanism of TS1 cellular phenotype

**Fig. 6:**
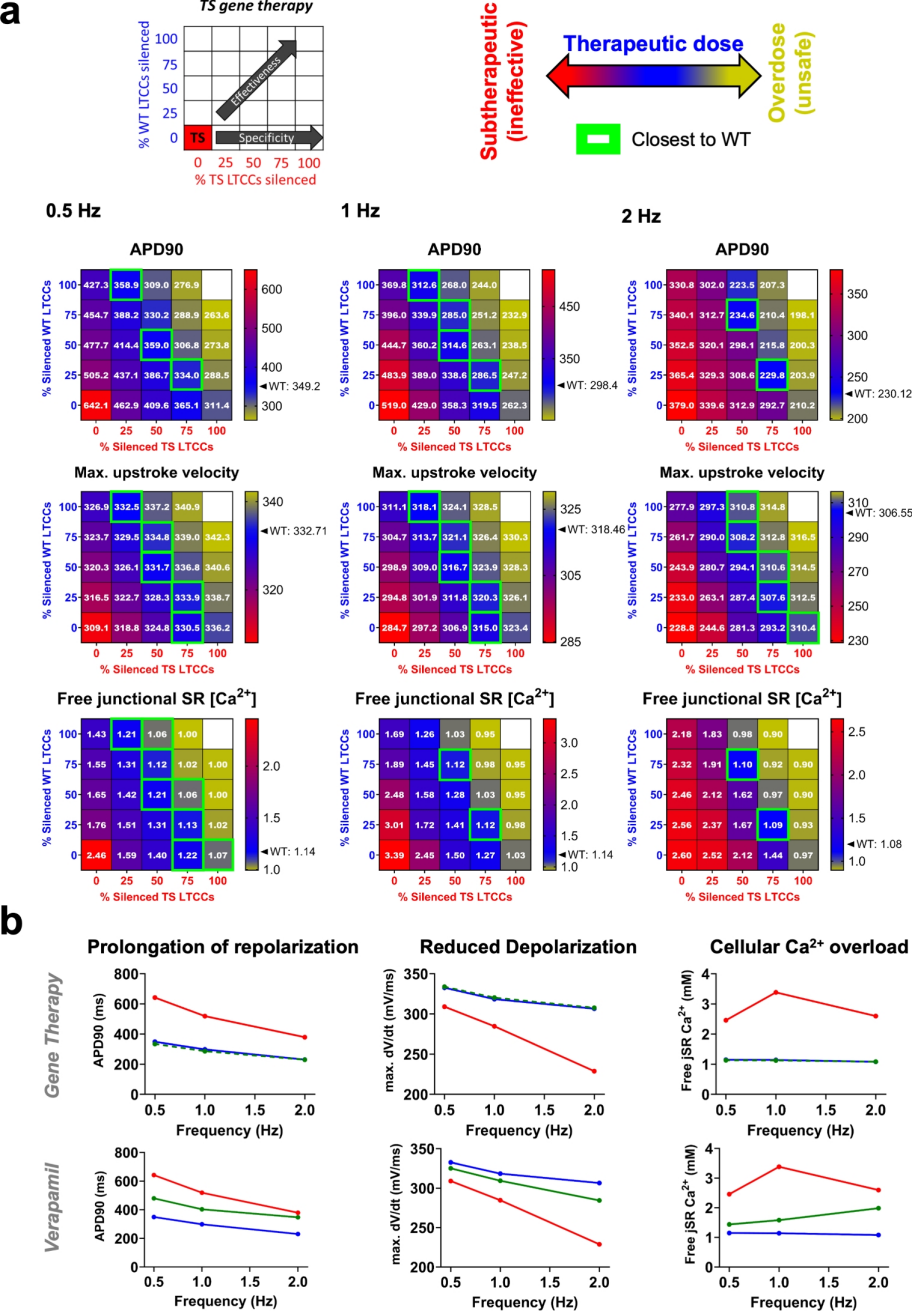
Simulated gene-silencing of LTCC in the TS1 ventricular model. (**A**) Top: schematic illustration of the simulation design. Simulations were conducted by varying the proportion of silenced TS1-LTCCs (*x*-axis) and silenced WT-LTCCs (*y*-axis). Color code represents different degrees of therapeutic efficacy, with red indicating subtherapeutic (ineffective), blue indicating effective and yellow indicating overcorrection of the phenotype. Green box is used to represent values closest to WT. Bottom: Simulations results at 0.5 Hz (left), 1 Hz (center) and 2 Hz (right) for the three evaluated phenotypes: APD90 (top row), maximum upstroke velocity (middle row), and jSR [$\hbox {Ca}^{2+}$]. (**B**) Comparison of the effects between the most-balanced gene therapy dosing (75% TS1-LTCCs and 25% WT-LTCCs silenced) (top) *vs.* non-specific targeting with LTCC blockers (bottom) on repolarization duration, depolarization reserve and cellular $\hbox {Ca}^{2+}$ overload. Blue, red and green represent WT, TS1 and TS1+treatment, respectively.

To target the primary mechanism of the TS1 phenotype, we considered two approaches: gene therapy (silencing) and pharmacological treatment with verapamil, an LTCC blocker tested in clinical settings with variable results^36,37^. We evaluated three TS1 phenotype aspects: AP elongation, upstroke velocity, and cellular $\hbox {Ca}^{2+}$ overload (free junctional SR [$\hbox {Ca}^{2+}$]) (Figure 6). Concerning gene silencing, we reasoned that RNA_i_s can variably silence WT and TS1 alleles with several degrees of specificity, as shown in^38^ for the case of ryanodine receptors. Therefore, we simulated the silencing of both WT and mutated LTCCs. Likewise, we explored different overall silencing levels, reflecting therapy effectiveness (e.g., varying Adeno-Associated Virus infection levels and RNA_i_ efficacy) (Figure 6A). The goal was to find a therapeutic dose that rescues the TS1 phenotype while maintaining safety (Figure 6A-B). The appropriateness of a given gene therapy dose/specificity was indicated by a color code, whereby red and violet indicate ineffective combinations (i.e. closest to TS1), blue indicates therapeutic value (i.e. closeness to WT, also indicated by green squares for visual aid) and yellow/gray indicates signs of overdose (i.e. overcorrected phenotype). The predictions yielded by the model showed that (Figure 6A) therapeutic silencing must be neither fully specific nor fully effective, but instead it requires a silencing close to 75% TS1 and 25% WT Ca_v_1.2 channels. This combination closely matches the WT phenotype at all tested frequencies. Additionally, a gene therapy of lesser specificity and slightly higher effectiveness, 50% TS1 and 75% WT Ca_v_1.2s silenced, was ranked second. Close examination of additional phenotypes in those 2 therapies (AP triangulation, AP and CaT shape and magnitude) demonstrated that the second-ranked gene therapy results in slowing of SR $\hbox {Ca}^{2+}$ release relative to WT and the first-ranked gene therapy (Supplementary Figure 8). Counter-intuitively, a fully specific and effective therapy (100% TS1 and 0% WT Ca_v_1.2s silenced) over-corrected the phenotype, likely due to excessive repolarizing currents over the remaining WT Ca_v_1.2s. Unexpectedly, gene therapies of reversed specificity (e.g. 0% TS1, 100% WT Ca_v_1.2s silenced) still yield a partial normalization of the phenotype at low stimulation frequencies. Finally, silencing a minimal percent (25%) of LTCCs, either WT, TS1 or combined, improved the phenotype at bradycardic frequencies but was ineffective at higher pacing rates.

The *in silico *administration of verapamil, modeled as per previously published methodology^31^, showed only partial correction of the cellular phenotype at low pacing rates, but not at high pacing rates (Figure 6B). Therefore, Verapamil’s effects were similar to an ineffective gene therapy dosage, while they were inferior when compared to the optimal gene therapy dose (75% TS1 + 25% WT silenced Ca_v_1.2s; Figure 6B). This may partially explain the unsatisfactory clinical outcomes of LTCC blockers^36,37,39^.

#### Secondary contributors to the TS1 cellular phenotype

**Fig. 7:**
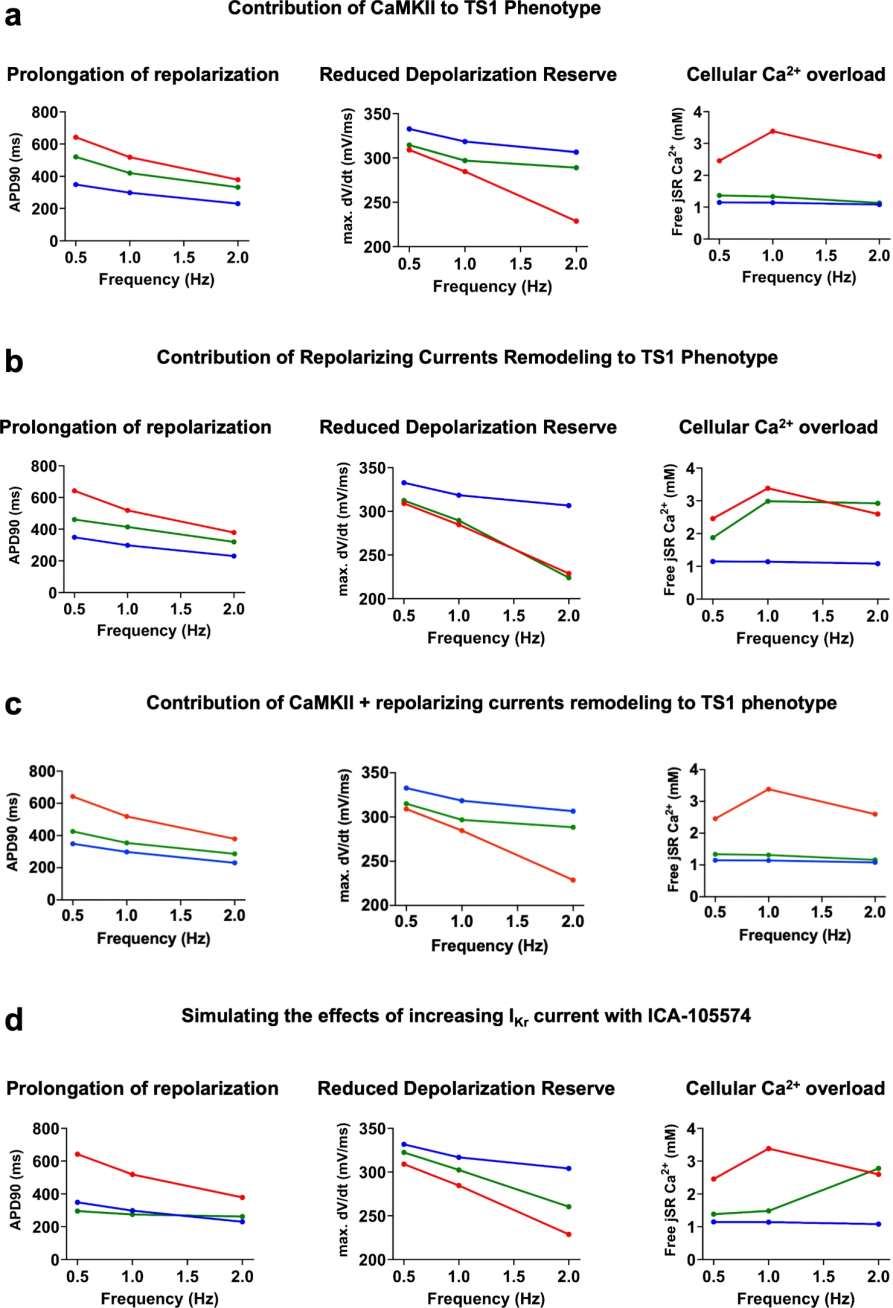
Role of secondary contributors to the TS1 cellular phenotype. (**A**) Contribution of CaMKII activation to TS1 phenotype assessed by simulating the effects of CaMKII inhibition (green) and comparing with TS1 (red) and WT (blue) on APD90 (left), upstroke velocity (center) and cellular $\hbox {Ca}^{2+}$ overload (right). (**B**) Contribution of altered repolarizing currents (40% I_Ks_ reduction and a +20.7 mV shift with 5% reduced conductance in the current-voltage relationship of I_to2_) to the TS1 cellular phenotype, simulated by restoring WT repolarizing currents in TS1 (green) vs. TS1 (red) and WT (blue). (**C**) Joint contribution of CaMKII activation and altered repolarizing currents to shaping the TS1 phenotypes (green) vs. TS1 (red) and WT (blue). (**D**) Simulated effects of increasing I_Kr_ current with ICA-105574 on the TS1 cellular phenotype. Details for the estimation of parameters for ICA-105574 simulation are presented in Supplementary Figure 9. Blue, red and green represent WT, TS1 and TS1+treatment, respectively.

Following the analysis of the primary contribution of mutated Ca_v_1.2 channels to the TS1 phenotype, we examined secondary contributors to the TS1 phenotype, including: 1) increased CaMKII activation due to $\hbox {Ca}^{2+}$ overload, leading to reduced I_Na_ and increased I_NaL_; 2) remodeling of repolarizing currents, specifically a 40% reduction in I_Ks_ (in the absence of $\hbox {Ca}^{2+}$ regulation and a +20.7 mV shift with 5% reduction in I_to2 _conductance). Although these mechanisms are experimentally identified^4^, their specific contributions to the TS1 phenotype need further clarification. As in the previous section, we assessed the TS1 phenotype at three levels: 1) repolarization prolongation; 2) depolarization reserve; 3) $\hbox {Ca}^{2+}$ overload. Figure 7A analyzed CaMKII’s role by simulating its inhibition through a 1000-fold increase in K_m_. This inhibition partially rescued AP prolongation and improved depolarization reserve, and almost completely rescued $\hbox {Ca}^{2+}$ overload, indicated by the free [$\hbox {Ca}^{2+}$] in the jSR. Figure 7B shows the impact of removing TS1-specific modifications to I_to2_ and I_Ks_ currents. This removal partially rescued AP prolongation, with minimal improvement in depolarization reserve and $\hbox {Ca}^{2+}$ overload. Also, we explored whether the simultaneous removal of the remodeling of repolarizing currents and of CaMKII activity would be able to rescue the TS1 phenotype. It was found (Figure 7C) that the TS phenotype was significantly ameliorated, although not normalized, highlighting the strong non-linearities immanent to the excitation-contraction coupling process alongside an important quantitative contribution of secondary phenotypes to the pathophysiology of this disease. Finally, to simulate a therapeutic approach counteracting the remodeling of repolarizing currents, we tested the effect of increasing I_Kr_ with an activator (ICA-105574), previously tested *in vivo*^4^. Based on experimental data, we introduced a +42 mV shift in inactivation, a −11 mV shift in activation, and a 2.3-fold increase in G_Kr _to fit our lab’s results on human ERG channels^32^ (Supplementary Figure 9). Figure 7D shows that I_Kr_ augmentation normalized APD90 and ameliorated, but did not completely restore, the depolarization reserve, in agreement with *in vivo* experiments. The impact on $\hbox {Ca}^{2+}$ overload was partially mitigated in a frequency-dependent manner, with negligible effects at high pacing frequencies. These findings highlight that while AP prolongation is significant in the TS1 phenotype, characteristics like reduced depolarization reserve and $\hbox {Ca}^{2+}$ overload are critical and differentially influenced by secondary contributors.

#### Contribution of increased INa_L_ to the TS1 phenotype

CaMKII-mediated enhancement of I_NaL_has been identified as a key contributor to AP prolongation in TS1^3,4^. To quantify this contribution, we simulated a theoretical condition of highly specific I_NaL_ blockade (95%). The model predicted that I_NaL_ accounts for approximately 16% (at 2 Hz) to 35% (at 0.5 Hz) of AP prolongation in TS1, and that this strategy does not correct for cellular $\hbox {Ca}^{2+}$ overload and the associated depolarization defects. We then assessed *in silico* the effects of two clinically available I_NaL _blockers, mexiletine and ranolazine, which have been used in TS patients with variable efficacy^3,37–41^. Simulations of virtual TS1 myocytes treated with these blockers (see Methods and Supplementary Figure 10) showed that mexiletine produced a partial positive effect on APD90 and intracellular $\hbox {Ca}^{2+}$ overload; however, it did not ameliorate the depolarization defect, in agreement with our i*n vivo *findings in a TS1 swine model^4^. In contrast, ranolazine had a neutral effect on all three phenotypes assessed (APD90, maximum dV/dt, and jSR $\hbox {Ca}^{2+}$), likely due to its relatively higher blockade of I_Kr_ compared to mexiletine and the partial I_Ca_block by mexiletine^33^. Such an effect on I_Ca_ could also explain the predicted higher AP shortening in mexiletine as compared to isolated 95% I_NaL_ block. Overall, our *in silico* TS1 model predicts that among the pharmacological interventions tested, the therapeutic benefit is greatest with the I_Kr_ activator ICA-105574, followed in order by verapamil, mexiletine, and ranolazine, consistent with the findings from our *in vivo *TS1 study^4^ .

### Arrhythmogenic potential of TS1 cells

**Fig. 8:**
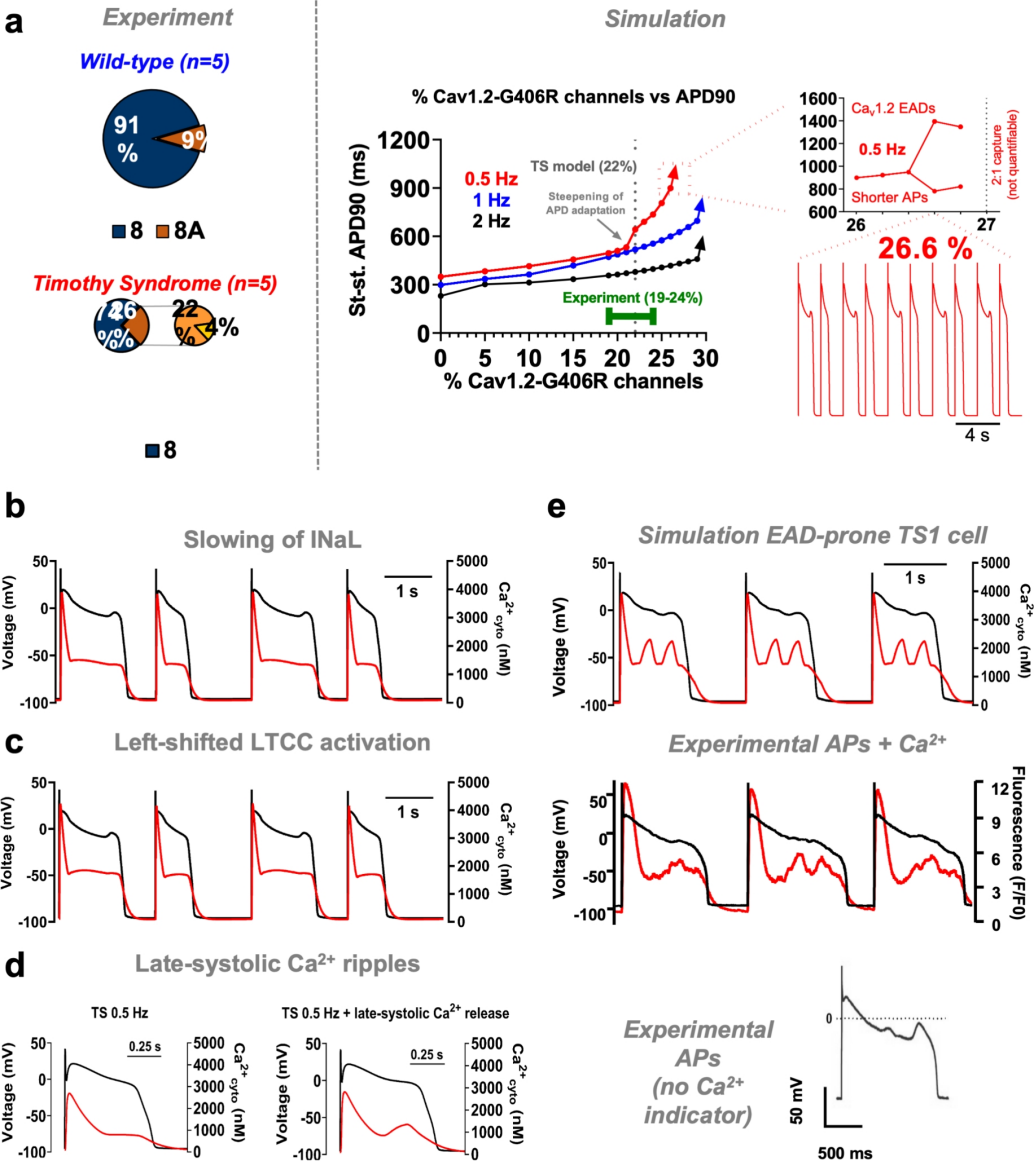
Arrhythmogenic Potential of TS1 Cardiomyocytes. (**A**) Left: experimental results showing increased expression of exon 8A in TS1 as compared to WT. Experimentally-derived average proportion of G406R-mutated LTCCs was 22%. Right: simulated relationship between increasing proportion of G406R-mutated LTCCs and APD90 at 0.5 Hz (red), 1 Hz (blue) and 2 Hz (black). Inset shows the behavior at 0.5 Hz when the proportion of G406R LTCCs exceeds 26%, characterized by appearance of EADs (starting at 26.6%) and 2:1 capture (starting at 27%). Green bar depicts the experimentally-observed range of G406R-mutated LTCCs. (**B**) Simulated traces of TS1 APs and CaTs at 0.5 Hz when introducing slowing of inactivation of the hl gate of I_NaL_ (tau-hl = 600 ms). (**C**) Simulated traces of TS1 APs and CaTs at 0.5 Hz when introducing 0.925 mV left-shift in Ca_v_1.2 activation. (**D**) Simulated traces of TS1 APs and CaTs at 0.5 Hz when introducing mathematical imposition of cytosolic $\hbox {Ca}^{2+}$ ripples/waves, emulating those caused by enhanced late-systolic SR $\hbox {Ca}^{2+}$ release. (**E**) Simulated traces of TS1 APs and CaTs at 0.5 Hz when introducing all the three aforementioned mechanisms and comparison with experimental data, both in the presence and absence of $\hbox {Ca}^{2+}$ fluorescent indicator. Experimental data and traces from Porta-Sanchez et al.^4^.

Regarding the arrhythmogenic substrate in TS1, our previous work demonstrated early afterdepolarizations (EADs) in approximately 25% of isolated swine ventricular myocytes from the TS1 *knock-in* model^4^. Here, we investigated the conditions for EADs occurrence, aiming to corroborate the model’s validity and to gain insights into EADs genesis, which are challenging to ascertain experimentally. We initially explored whether increasing the proportion of mutated-LTCCs would enhance EADs susceptibility. Varying the proportion of TS1-LTCCs, we observed a linear rise in APD90 up to 21% mutated channels, consistent with the lower range of experimental findings (22±2%, range 19%−24%) (Figure 8A). Higher mutation levels than the experimental range (19–24%) induced steep APD90 increases, leading to APD alternans and Ca_v_1.2-mediated EADs at 0.5 Hz (>26.6%) and 2:1 capture (>27%). Next, we investigated additional mechanisms potentially contributing to EADs in TS1. Literature suggests EADs may arise from: i) slowed kinetics of I_NaL_^42^; ii) left-shifted LTCC activation^43^; and iii) cytosolic $\hbox {Ca}^{2+}$ ripples due to enhanced late-systolic SR $\hbox {Ca}^{2+}$ release with ensuing I_ti_^4^. Slowing the inactivation of the hl gate of I_NaL_ resulted in prolonged AP and increased intracellular Na^+^ and $\hbox {Ca}^{2+}$ (Figure 8B). This alteration led to EADs at a time constant of 600 ms, phenomena not observed in WT cells even with an increase in time constant twice as large (Supplementary Figure 11). Secondly, TS1 cells were notably more susceptible to EADs (*vs.* WT) caused by leftward shifts in Ca_v_1.2 activation (Figure 8C), requiring only −0.925 mV *vs.* −4.5 mV in the WT (Supplementary Figure 11). Third, experimental evidence indicated that EADs could also be associated with increased late-systolic SR $\hbox {Ca}^{2+}$release, a finding also reproduced by the TS1 model (Figure 8D). Finally, Figure 8E illustrates the behaviour of a simulated TS1 cell (22% G406R channels) where all three mechanisms coexist, closely resembling experimental traces from isolated myocytes of the TS1 swine model^4^. Lastly, we assessed whether targeting primary and secondary mechanisms could mitigate EAD-prone TS1 cell arrhythmogenicity (Supplementary Figure 12). Gene therapy effectively normalized the TS1 phenotype despite EADs susceptibility. I_Kr_ activation prevented EADs and reduced APD, yet did not prevent cellular $\hbox {Ca}^{2+}$ overload. CaMKII inhibition mitigated cellular $\hbox {Ca}^{2+}$ overload and prevented EADs occurence, despite only partial correction of AP prolongation.

In conclusion, our *in silico* findings underscore that simulated TS1 cells are more susceptible to multiple EADs-generating mechanisms as compared to WT cells, while assessing the potential of targeted therapies to mitigate their arrhythmogenic potential.

## Discussion

### Summary of key findings 

This study presents a novel mathematical model of swine ventricular action potential and $\hbox {Ca}^{2+}$handling. Adapted from a state-of-the-art human ventricular model^17^, our model incorporates species-specific details using extensive experimental data from our laboratory^4^. It accurately replicates the complex electrophysiological features of TS1 identified in the first-ever *knock*-*in* large mammalian (swine) model^4^, departing in this respect from other models of the disease^10,44–48^. Specifically, our model faithfully reproduces not only the pronounced AP prolongation characteristic of TS1, but also crucial aspects such as intracellular $\hbox {Ca}^{2+}$ overload-induced modifications like CaMKII-mediated changes in I_Na_ and I_NaL_, and alterations in repolarizing currents, while retaining key characteristics of its parent models such as CaMKII regulation of $\hbox {Ca}^{2+}$handling fluxes^17 ^and Calcium-Dependent Facilitation^21^. This comprehensive simulation allows us to differentiate between primary and secondary contributors to the TS1 phenotype, enhancing our understanding of its pathophysiology. Moreover, leveraging known mechanisms of EADs, our simulations confirm that TS1 cells are significantly more susceptible to these abnormalities compared to WT cells, reflecting their altered balance of inward and outward currents. Ultimately, our *in silico *TS1 model holds promise for identifying novel therapeutic targets at the cellular level, potentially paving the way for targeted interventions aimed at mitigating the arrhythmogenic risks associated with TS1.

### Advancing swine ventricular myocyte modeling: inter-species variation 

In the past three decades, mathematical models of ventricular cardiomyocytes have been developed for various species, including guinea pigs^9–11^, rabbits^12–14^, dogs^15,16^, and humans^17–19,21^. Despite the essential role of porcine models in cardiovascular research^8,49^, the first swine-specific mathematical model was introduced recently in 2021^20^(Supplementary Figure 1). Porcine models are widely used in preclinical studies, yet their application in cellular electrophysiology has been relatively limited. Leveraging extensive experimental data from characterizing a TS1 knock-in porcine model^4^, we developed a new swine ventricular myocyte model, validated against a wide range of AP and $\hbox {Ca}^{2+}$ transient characteristics.

Using a methodology proposed by Sobie and colleagues^26^, we performed a sensitivity analysis across 500 random model variations, aimed to assess the model’s robustness by varying key parameters reflecting biological stochasticity. It also compared findings with the parent human ORd model, highlighting differences in the influence of major ionic fluxes on AP configuration and intracellular $\hbox {Ca}^{2+}$ dynamics. Notably, our analysis revealed physiological differences between swine and human cardiomyocytes. Unlike the human ORd model, where I_Kr_ is the major determinant of AP repolarization, our swine model indicates that repolarization reserve relies more heavily on I_Ks_, consistent with prior experimental^4,50^and theoretical^20^ studies showing lower I_Kr _density in swine than in humans. Interestingly, parameters such as maximal SERCA pump activity, dissociation constant, and maximum NCX conductance exerted a more pronounced impact on AP kinetics in swine than in human. On what concerns the TS1 swine model, the sensitivity analysis revealed a stronger dependency of P_Ca_ in APD90 *vs.* its WT counterpart, plus a mild negative impact of P_Ca_ on max. dV/dt, consistent with the emerging notion that upstroke velocity can be influenced in a $\hbox {Ca}^{2+}$and CaMKII-dependent manner^4^. In the above context, interspecies differences in excitation-contraction coupling mechanisms pose challenges for translating findings from animal models to humans. Sobie’s regression-based approach has facilitated quantitative translation of electrophysiological responses across species, from rabbits and mice to humans^27^, with Gaur et al. recently initiating the first swine-to-human *in silico *comparison^20^. Our sensitivity analysis supports these efforts, demonstrating the feasibility and importance of translating electrophysiological responses from swine to humans, highlighting the opportunity in future studies to implement this methodology to derive predictors that can accurately translate measured electrophysiological responses from swine to human.

Regarding murine-to-swine translability on what concerns TS1, our in silico AP-clamp data are in line with experimental findings from the Santana laboratory^34^. Our results, either from our experimental swine study^4^ and now *in silico* (this study), suggest that fundamental aspects of TS1 are conserved between small rodents and swine, such as cellular $\hbox {Ca}^{2+}$overload^34^, a non-obvious phenotypic TS1 dependency on the AP waveform^34^and a prominent role of CaMKII^2^. However, other disease characteristics differ between small and large mammals: TS1 mice^34 ^and rats^2 ^predominantly exhibit DADs as arrhythmogenic mechanism, whereas large mammals like swine show a prevalence of EADs^4^. Kinetically, the Anderson laboratory^2^ demonstrated in TS1 rat myocytes that CaTs are larger than in WT cells and have a shorter FDHM (peakier), akin to the initial phase of CaTs in our swine model, which is also of higher magnitude. However, the late plateau phase in CaTs appears unique to large mammals, due to their APs as compared to rodents.

### Novel insights: how many mutated LTCCs in a TS1 cardiomyocyte? 

Experimental data in this study predominantly derive from recent characterization of a TS1 *knock-in*swine model^4^, supplemented by newly collected data critical for understanding TS1. Hitherto, quantifying mutated LTCCs in TS1 relied on assumptions from Splawski et al.^1^, estimating a 11.5% mutation rate based on a 23% exon 8A inclusion in humans assuming heterozygosity for the p.Gly406Arg mutation. However, our previous work in heart^4 ^and a recent study on TS1 neurons^51^ questioned the validity of a 50% WT:50% mutated assumption due to observed differences in exon 8A inclusion ratios between WT and TS1. In this study, we quantified the higher exon 8A inclusion in TS1 hearts compared to WT counterparts (26% vs. 9%), with mutated exon 8A accounting for 22% (range 19%−24%) of LTCCs in TS1. This proportion exceeds previous estimates, underscoring the need for further investigation into the mechanisms driving increased exon 8A inclusion and its potential clinical implications. Of note, at the time of performing the simulations varying the percentage of TS1-LTCCs (Figure 8A), our experimental team had only provided our *in silico* team the average proportion of TS1-LTCCs. Interestingly, our model predicted distinct disease phenotypes precisely within the experimental range of exon 8A expression (19%−24%). These predictions included progressive AP prolongation with increasing mutated channels, steepening of APD rate-adaptation, development of EADs at 26.6%, and 2:1 conduction block at 27%, faithfully recapitulating TS1 characteristics. Notably, p.Gly406Arg-Ca_v_1.2 parametrization was solely based on voltage-clamp data, with no AP or CaT characteristics taken as part of the training set (i.e. APD in TS1 is a model prediction). This reciprocal validation between theory and experiment strengthens both sets of findings. We believe that this cross-fertilization between theory and experiment provides robustness to both findings simultaneously. Combined, our theoretical and experimental findings suggest that developing new tools to tilt the exon 8A inclusion ratio towards the WT allele, even if slightly, might result in a non-linear improvement in the TS1 phenotype, highlighting the need for further investigation.

### In Silico TS1 swine model: unveiling pathophysiological mechanisms and therapeutic targets

After developing and validating the TS1 swine model, translational relevance of the model was investigated by: i) dissect primary and secondary contributors to the TS1 phenotype, such as intracellular $\hbox {Ca}^{2+}$ overload and repolarizing current remodeling; ii) demonstrate the arrhythmogenic potential of TS1 cells; iii) evaluate the model’s ability to predict pharmacological targets for restoring the phenotype and reducing propensity to EADs. Our analysis of primary and secondary contributors to the TS1 phenotype provided crucial insights for clinical translation. The model predicts that normalizing AP duration alone, without addressing the primary defect in $\hbox {Ca}^{2+}$ channels, fails to normalize the cellular phenotype, particularly failing to fully correct cellular $\hbox {Ca}^{2+}$ overload and associated depolarization abnormalities. This distinction from other forms of long QT syndrome underscores TS1’s higher clinical malignancy.

Furthermore, the model identified potential therapeutic targets: gene silencing to counteract Ca_v_1.2 mutation effects, CaMKII inhibition to mitigate $\hbox {Ca}^{2+}$ overload and slowed AP upstroke velocity, and I_Kr_ current activation to shorten AP by enhancing repolarizing currents. A ranking of these therapeutic strategies emphasizes that correcting the primary defect through gene-silencing is the preferred therapeutic strategy. Other approaches only partially correct the complex cellular phenotype, despite AP shortening. Also, we have shown how the *in silico *model may serve as a platform to optimize the necessary balance between silencing potency and specificity for mutated LTCCs, aiming for the best therapeutic silencing dose. Interestingly, the predicted benefits of gene therapy relative to verapamil are very significant, especially when considering recent discoveries^39 ^which show that reducing channel inactivation (i.e. the cause of TS1) leads to a loss of use-dependent block by verapamil, resulting in a lack of therapeutic efficacy^36,37^.

Regarding TS1’s arrhythmogenic potential, the model predicts TS1 cells are markedly more susceptible to generating EADs compared to WT cells, consistent with swine* in vitro *data^4^. *In silico* TS1 myocytes also exhibit slowed AP upstroke velocity, aligning with experimental findings in isolated swine TS1 myocytes and functional reduced depolarization reserve *in vivo *^4^. Together, these aspects highlight the model’s ability to accurately replicate TS1’s high arrhythmogenic risk. Importantly, in simulations of TS1 cells highly prone to EADs, therapy differences were magnified. Gene therapy, I_Kr_ activation and CaMKII inhibition prevented EADs, but to different extents. Gene therapy, achieving complete TS1 phenotype rescue despite extreme EADs susceptibility, retained the top therapeutic rank. In contrast, I_Kr_ activation incompletely prevented cellular $\hbox {Ca}^{2+}$ overload, ranking second. CaMKII inhibition prevented EADs and partially alleviated cellular $\hbox {Ca}^{2+}$ overload, but with only a partial correction of AP prolongation. Moreover, these therapies, tailored for worst-case scenarios (extreme EAD susceptibility), are anticipated to prevent EADs in less severe instances. As an example, Supplementary Figure 13 shows that CaMKII inhibition ceases EADs and reduces cellular $\hbox {Ca}^{2+}$ overload when only 1 EAD-generating mechanism (left-shift of LTCC activation) is active in the cell.

### Limitations

Our model is not devoid of limitations. Firstly, its training set comprised data from a range of stimulation frequencies (0.5–2Hz) that did not include extreme tachycardia. Secondly, like its parental ORd model^17^, our model associates SR $\hbox {Ca}^{2+}$ release with I_Ca_ magnitude. This linkage, effective in WT swine models, results in a somewhat exaggerated increase in CaT amplitude in TS1 compared to WT cells due to heightened I_Ca_ during the AP. Likewise, stemming from the same I_Ca_-J_rel_ coupling, the lack of a significant I_Ca_ during diastole leads to a quantitatively unimportant RyR2-dependent diastolic $\hbox {Ca}^{2+}$ leak, which precludes the *in silico* study on the role of CaMKII-dependent RyR2 phosphorylation on what concerns SR $\hbox {Ca}^{2+}$overload in TS1. Nonetheless, rigorous training and validation ensure accurate replication of CaT kinetics, AP features, and critical CaMKII-mediated effects across phenotypes, supporting its utility for single-cell research and future incorporation in tissue/organ-scale simulations. Additionally, as in the parent models (ORd 2011 and BPS2020)^17,21^, the I_Ca_ I-V curve does not perfectly fit the positive potentials and this remains the case for our novel swine model. This could lead to mild I_Ca_ underestimation at positive potentials. Lastly, our experimental myocyte data did not show spontaneous overload-induced SR $\hbox {Ca}^{2+}$release in either phenotype up to 2 Hz^4^, preventing inclusion of such a module in our model. Future directions include the integration of a PKA module, which will be validated as detailed data from large white swine models become available, particularly in cells stimulated with beta-adrenergic agonists. This module will be crucial for evaluating the effects of beta-blocker therapy in TS1.

## Conclusions

In summary, we have developed and validated a novel mathematical model of Large-White swine ventricular myocyte, leveraging extensive experimental data^4^. Our findings underscore the potential of this model as a versatile tool for studying cardiac electrophysiology in physiological and pathological conditions, such as TS1. Moreover, our study highlights the capability of this model to serve as a robust platform for investigating therapeutic targets in TS1. Given the limited availability of electrophysiological data and mathematical models for pigs, we anticipate that our model will not only advance TS1 research but also facilitate broader investigations into arrhythmogenesis in swine models of cardiac diseases.

## Supplementary Information

Supplementary Information.

## Data Availability

All data needed to evaluate the conclusions in the paper are present in the paper and/or the Supplementary Materials. MatLab code of the novel swine model is available at the following link: https://shorturl.at/tQiRN.
