# Supplementary material for: A novel computational model of swine ventricular myocyte reveals new insights into disease mechanisms and therapeutic approaches in Timothy Syndrome
Source: Sci Rep. 2024 Nov 30;14:29792. doi: 10.1038/s41598-024-80726-2 (PMC11608236; doi:10.1038/s41598-024-80726-2)
Supplement: Supplementary file 1 — Supplementary Information. [file 41598_2024_80726_MOESM1_ESM.pdf]

Supplementary Material to:

A Novel Computational Model of Swine Ventricular Myocyte Reveals New Insights into Disease Mechanisms and Therapeutic Approaches in Timothy Syndrome

Alessandro Trancuccio[1,2], Carmen Tarifa[3], Rossana Bongianino[1], Silvia G. Priori[1,2,3], and Demetrio J. Santiago[3*]

[1]Molecular Cardiology Unit, IRCCS Istituti Clinici Scientifici Maugeri, Pavia, Italy
[2]Department of Molecular Medicine, University of Pavia, Pavia, Italy
[3]Novel Arrhythmogenic Mechanisms Program, Centro Nacional de Investigaciones Cardiovasculares (CNIC), Madrid, Spain
[*]Corresponding Author; email: demetriojulian.santiago@cnic.es

Supplementary Figures

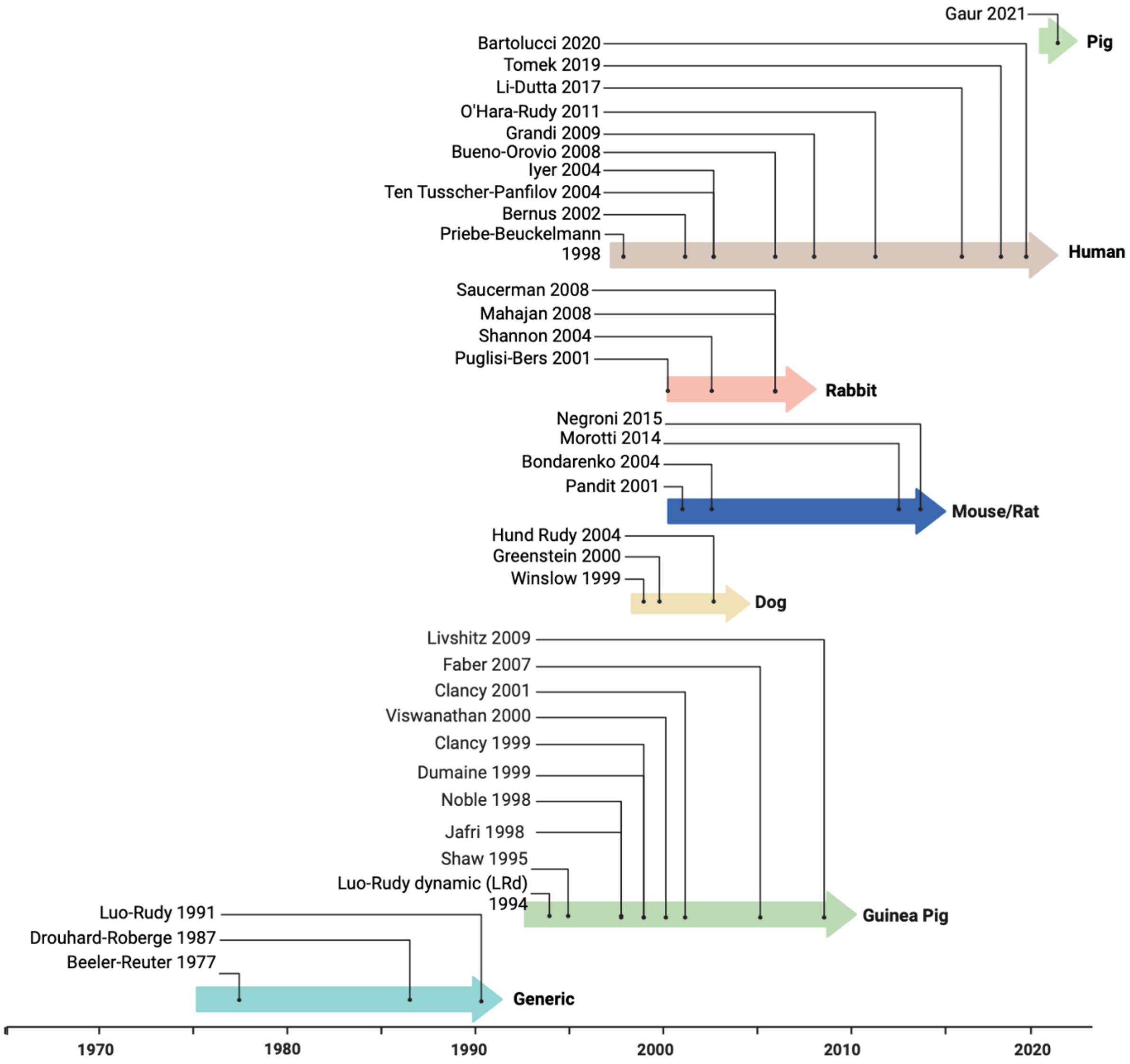

Figure S1. Timeline of main mathematical models of ventricular myocytes developed for different species. The figure summarizes the main mathematical models developed over the years for different species, including generic models, guinea pig models, canine models, mouse/rat models, rabbit models, human models. As depicted in the top right corner of the figure, it wasn't until 2021 that the first and only computational swine model was developed.

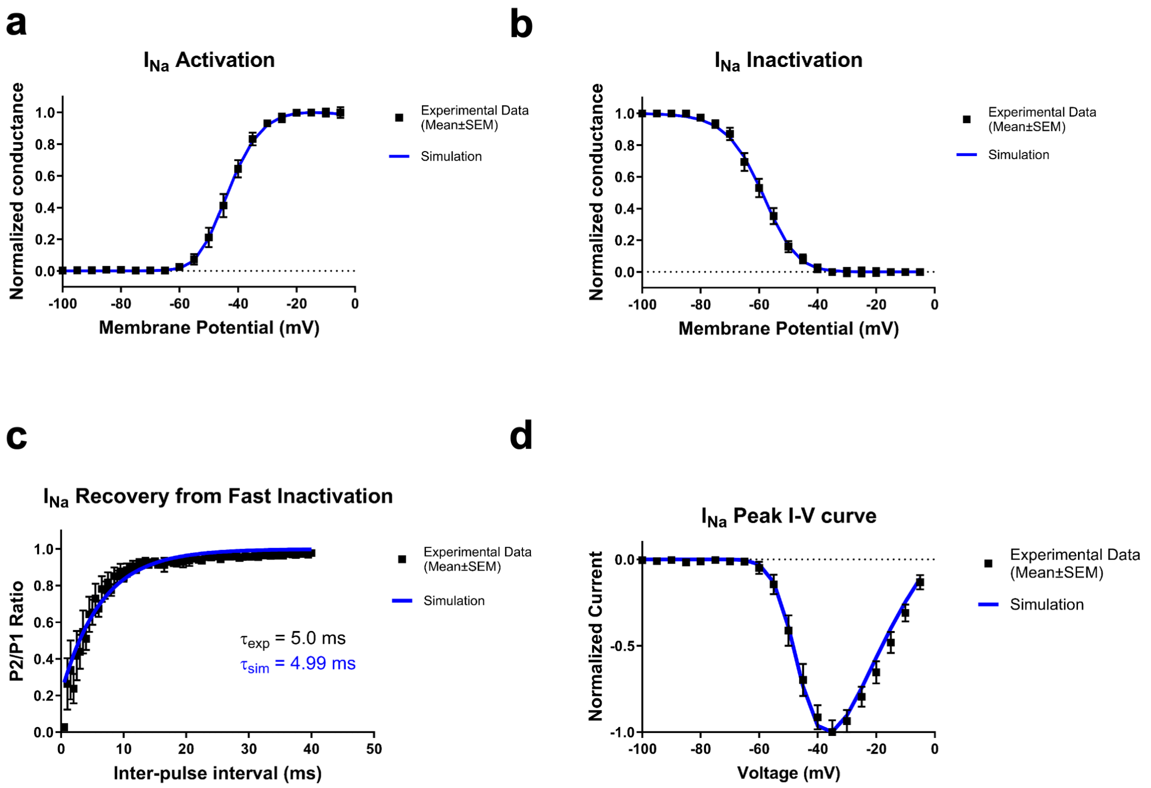

Figure S2. Fitting of WT Na^+gating parameters. Comparison of the experimental (black, mean±SEM) and simulated (blue) data for I_{Na} activation (A), inactivation (B), recovery from inactivation (C) and peak I-V relationship (D). Experimental data from Porta-Sanchez et al.[1]

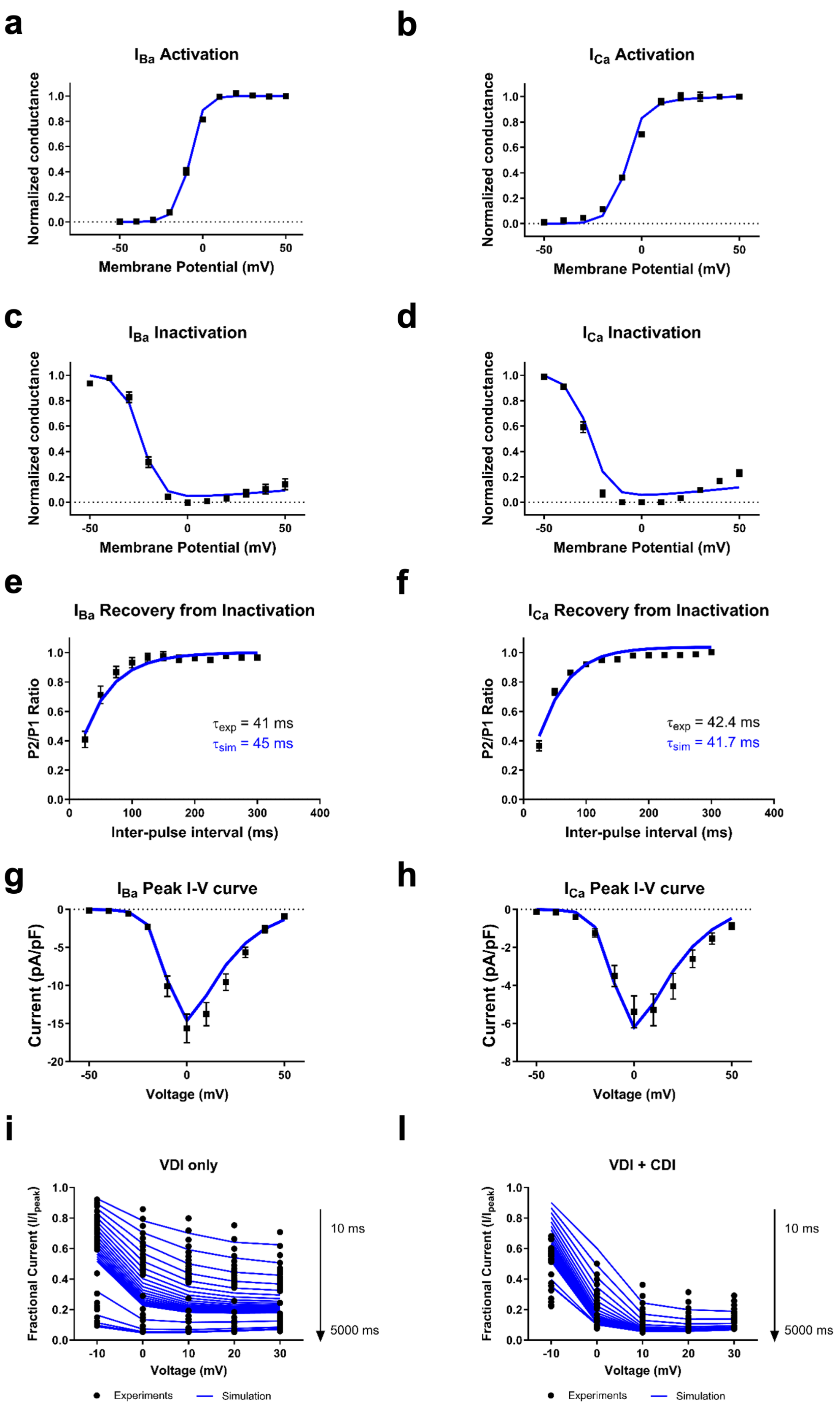

Figure S3. **Fitting of WT I_{Ba} and I_{Ca} gating parameters.** Comparison of the experimental (black, mean±SEM) and simulated (blue) data for I_{Ba} and I_{Ca} activation (A-B), inactivation (C-D), recovery from inactivation (E-F), peak I-V relationships (G-H) and fractional remaining current (FRC) (I-L). Experimental data from Porta-Sanchez et al.[1]

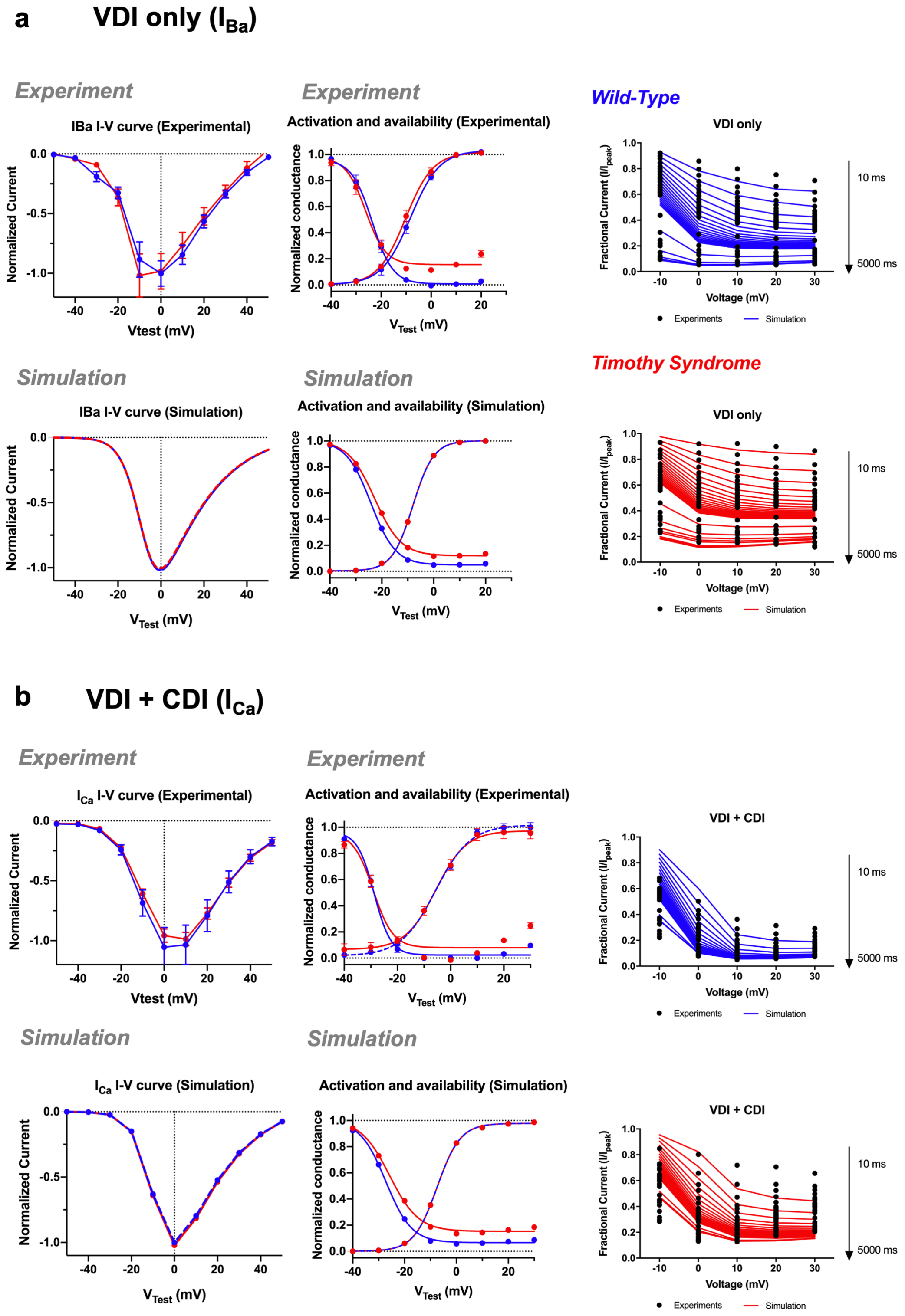

Figure S4. Fitting of TS1 I_{Ba} and I_{Ca} gating parameters. (A) Comparison of the experimental (top) and simulated (bottom) I_{Ba} currents (VDI only) in WT (blue) and TS1 (red). Peak I-V relationships are shown on the left, activation and availability are shown on the center and FRC are shown on the right. (B) Comparison of the experimental (top) and simulated (bottom) I_{Ca} currents (VDI+CDI) in WT (blue) and TS1 (red). Peak I-V relationships are shown on the left, activation and availability are shown on the center and FRC are shown on the right. Experimental data from Porta-Sanchez et al.[1]

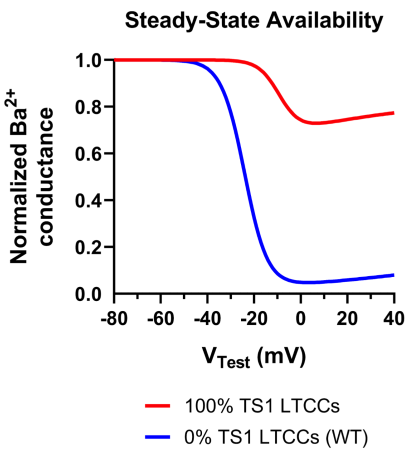

Figure S5. Ba^{2+} current steady-state availability with 100% G406R-LTCCs. Simulation of the voltage-dependence of Ba^{2+} current inactivation with 100% TS1-LTCCs (red) and comparison with WT (blue). The simulations qualitatively reproduced the experimental data obtained in CHO cells by Splawski et al.[2] (Refer to Figure 5-I in Splawski et al.[2] for comparison).

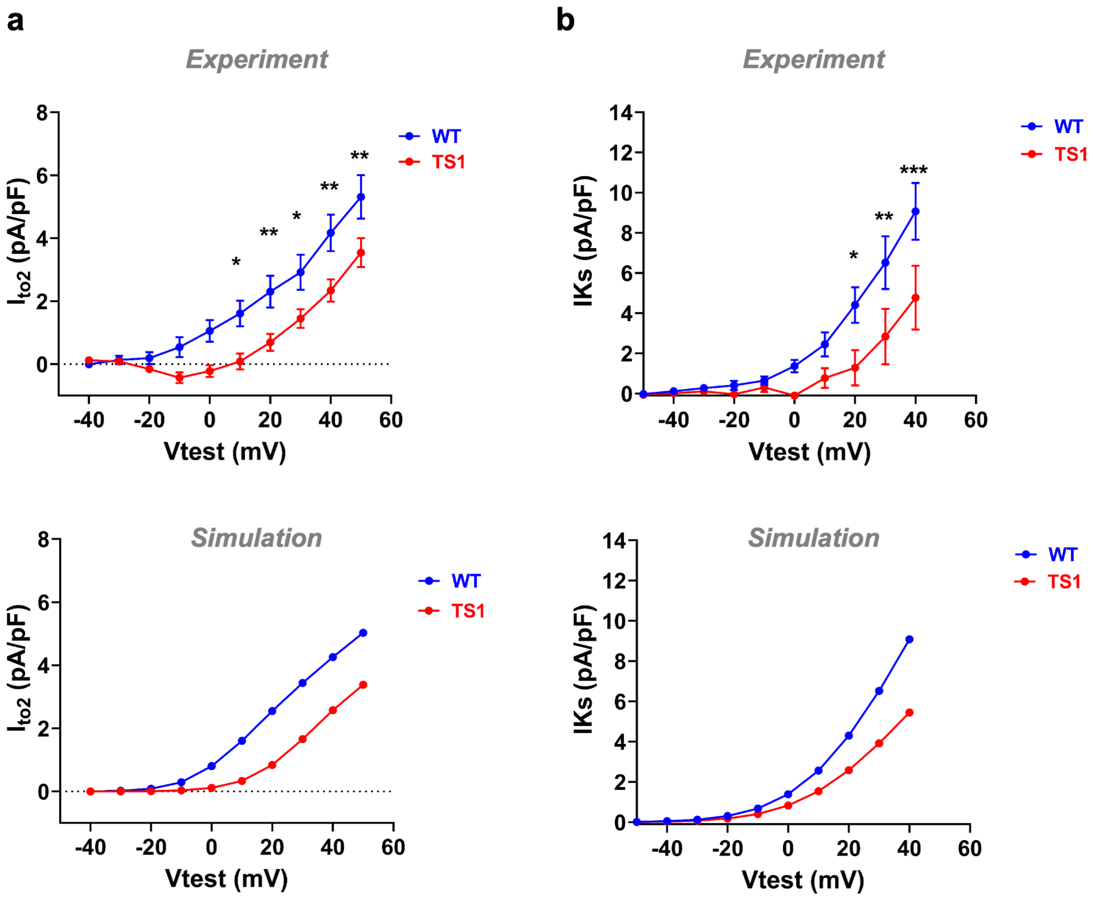

Figure S6. Remodeling of repolarizing currents in TS1. (A) Comparison of peak I-V relationships of experimental (top) and simulated (bottom) I_{to2} current in WT (blue) and TS1 (red). (B) Comparison of peak I-V relationships of experimental (top) and simulated (bottom) I_{Ks} current in WT (blue) and TS1 (red), in the absence of Ca^{2+} regulation. Experimental data for I_{Ks} from Porta-Sanchez et al.[1]

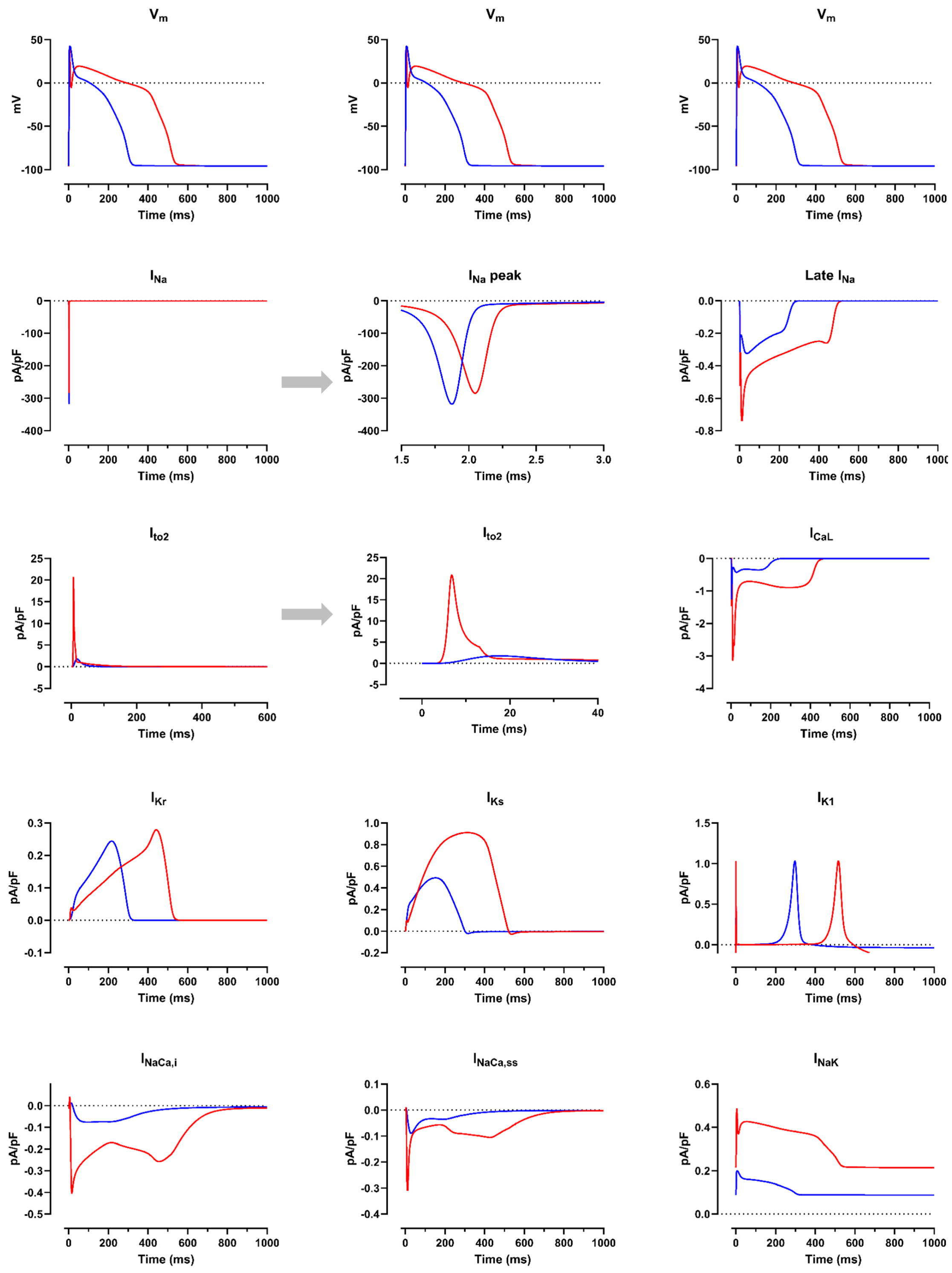

Figure S7. Comparison of simulated currents between WT (blue) and TS1 (red) pig ventricular myocytes during paced action potentials at 1 Hz. (Top Row) Simulated APs, repeated in each column for timing purposes. Lower Rows (left to right, top to bottom): I_{Na}, peak I_{Na} detailed time course, late I_{Na}, I_{to2}, I_{to2} detailed time course, I_{CaL}, I_{Kr}, I_{Ks}, I_{K1}, $I_{NaCa,i}$, $I_{NaCa,ss}$, and I_{NaK}.

TS1 + Gene therapy: additional phenotypes examined

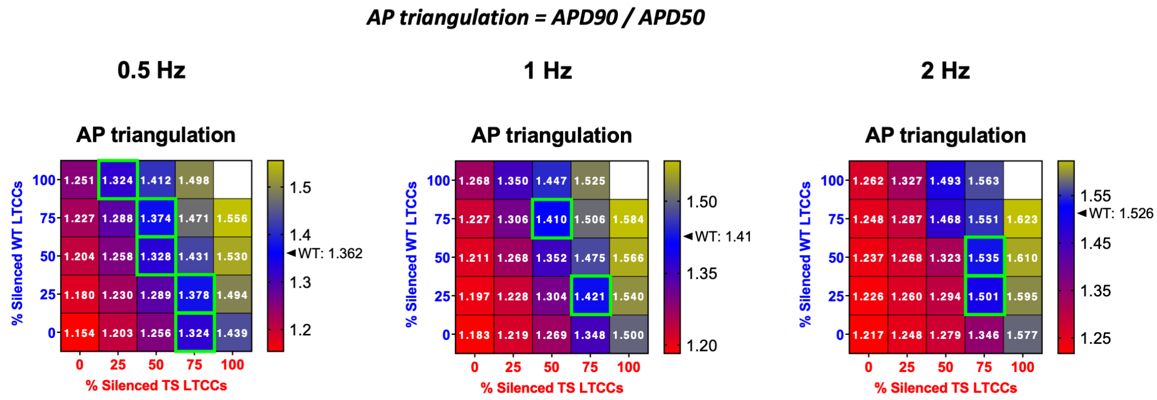

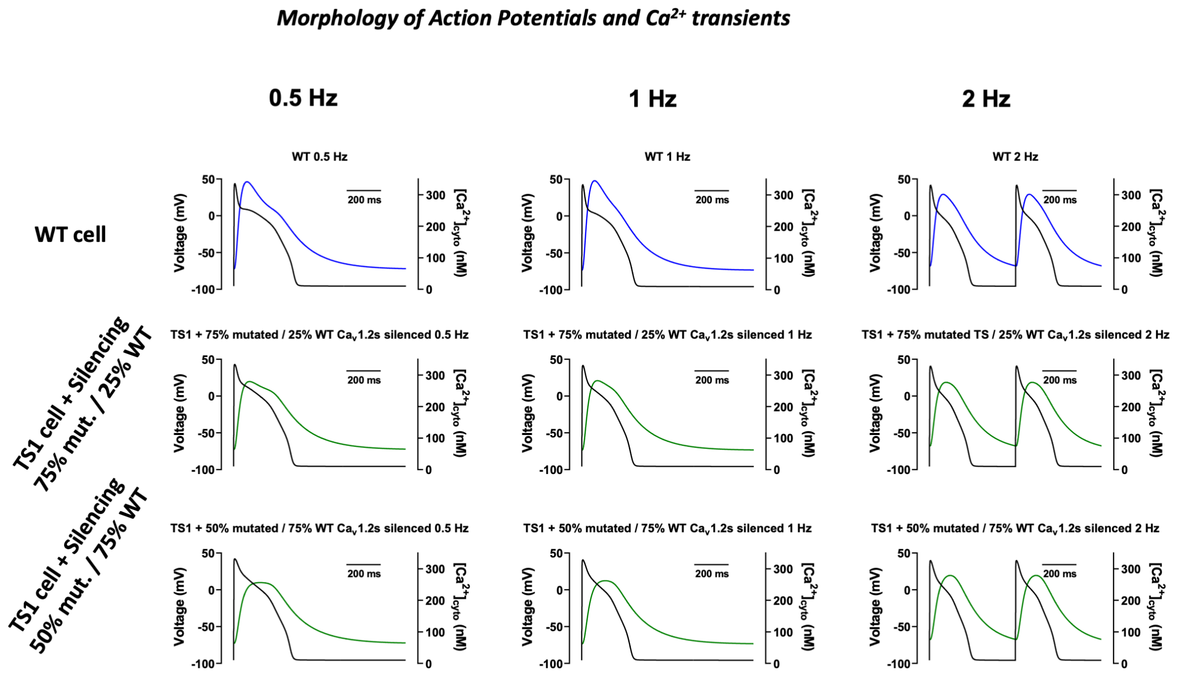

Figure S8. **Simulated gene-silencing of LTCC in TS1: additional phenotypes examination.** *Top panel*: Simulations results for different combination of WT/TS1 LTCCs silencing at 0.5 Hz (left), 1 Hz (center) and 2 Hz (right) on AP triangulation (APD90/APD50). Same color scheme used in Figure 6. *Bottom panel*: Comparison of the effects between two combinations of LTCC silencing (75% TS1 and 25% WT silenced *vs.* 50% TS1 and 75% WT silenced) on the morphology of action potential and Ca^{2+} transient at 0.5 Hz (left), 1 Hz (center) and 2 Hz (right).

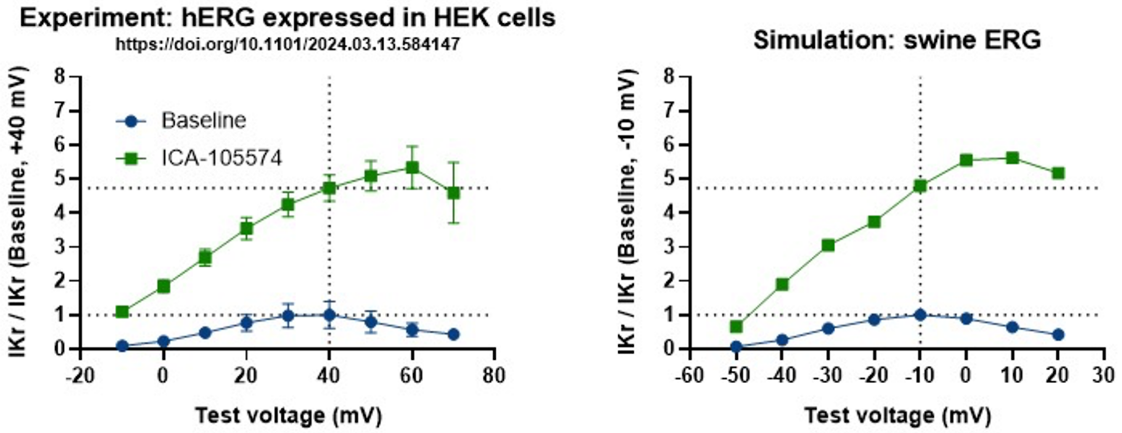

Figure S9. Simulation of the changes produced by ICA-105574 on the I_{Kr} I-V relationship. Experimental effects of ICA-105574 on I_{Kr} I-V relationship in HEK cells expressing hERG channels (left panel, data from[3]) and comparison with simulated effects on I_{Kr} in the swine cellular model. ICA-105574 treatment was simulated by +42 mV inactivation shift, -11 mV activation shift and 2.277-fold increase in I_{Kr} conductance (G_{Kr}).

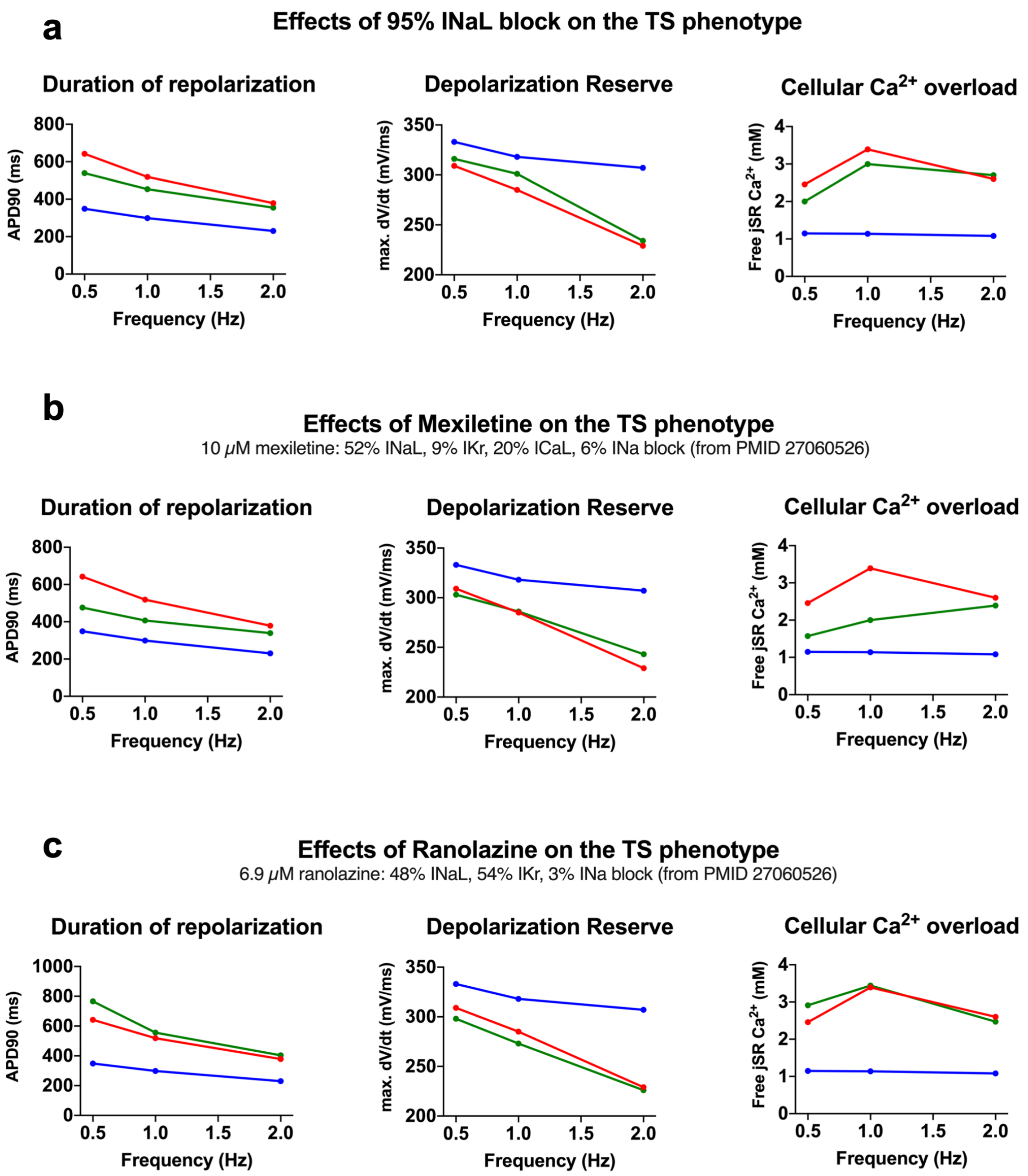

Figure S10. Contribution of Increased I_{NaL} to the TS1 Phenotype. (A) Simulation of a theoretical condition of highly specific I_{NaL} blockade (95%). (B) Simulation of the effects of 10 μM mexiletine on the TS1 phenotype. (C) Simulation of the effects of 6.9 μM ranolazine on the TS1 phenotype. For all the graphs blue indicates WT, red untreated TS1 and greeen treated TS1.

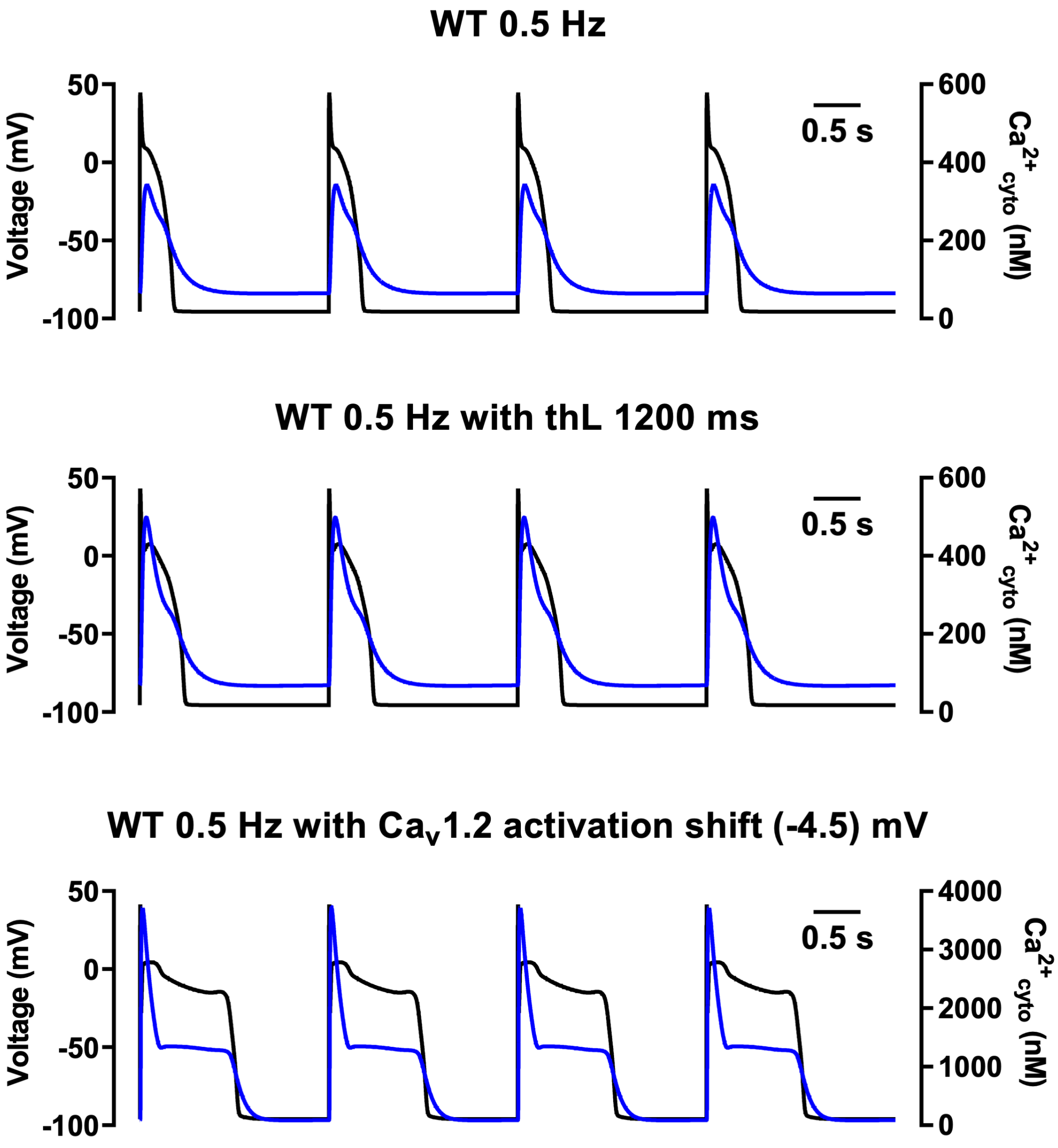

Figure S11. Susceptibility of WT myocytes to known EADs-generating mechanisms. Slowing the inactivation of the *hl* gate of I_{NaL} up to a time constant of 1200 ms in the *in silico* WT cardiomyocyte resulted in prolongation of AP, without generation of EADs. On the contrary, leftward shifts in LTCC activation by 4.5 mV determined massive AP prolongation and EADs generation.

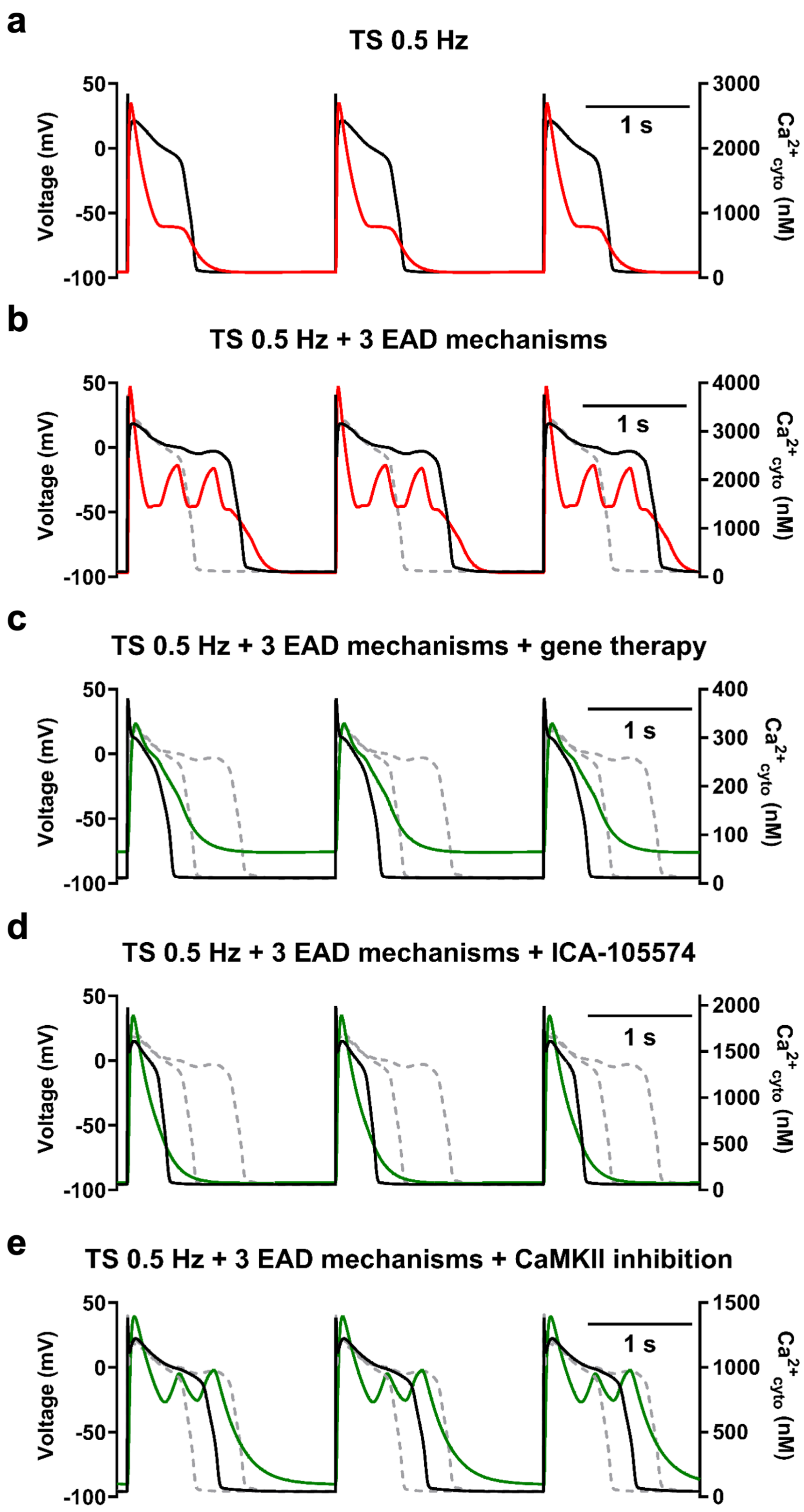

Figure S12. **Effects of therapeutic interventions when applied to a TS1 cell of extreme propensity to EADs.** Simulated APs and CaTs at 0.5 Hz steady-state in a TS1 cell (A) and in a TS1 cell presenting an extreme propensity to EADs, obtained by combination of 3 EADs-generating mechanisms (B). In the panels below the simulated effects of gene therapy (C), I_{Kr} activation with ICA-105574 (D) and CaMKII inhibition (E). The dashed gray APs in panels (B-E) are aimed for visual comparison, and indicate the APs of the TS1 cell with and without a propensity for EADs.

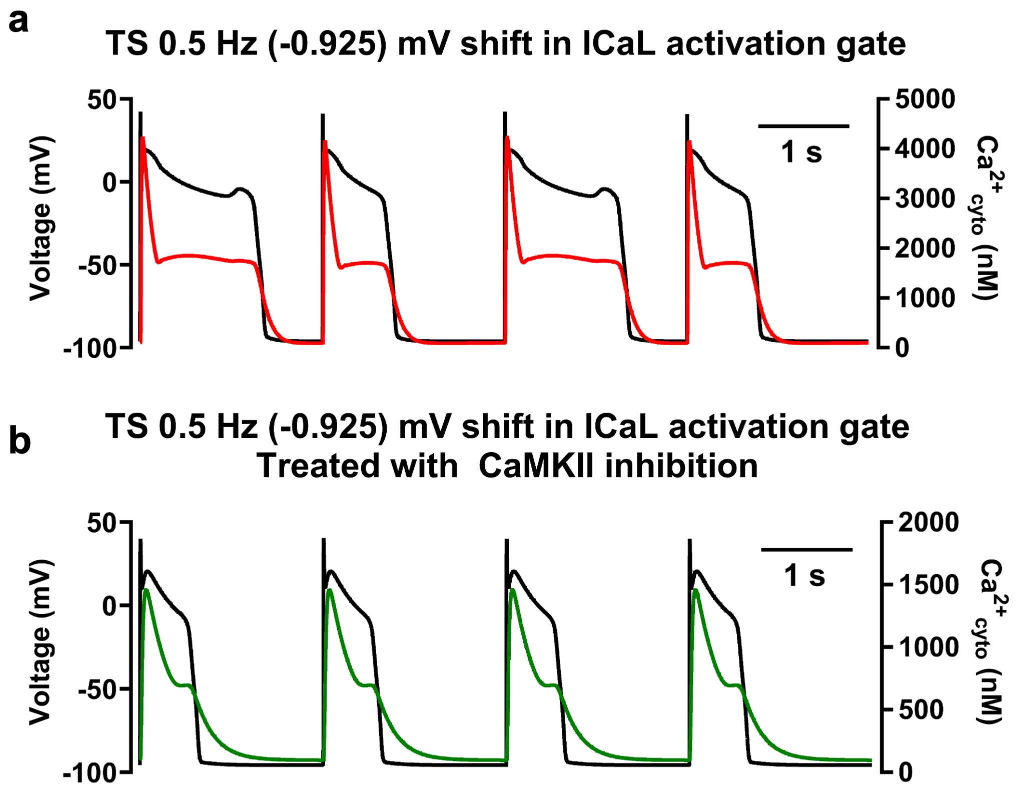

Figure S13. Effects of CaMKII inhibition when applied to a TS1 cell with moderate propensity to EADs. Simulated traces of TS1 APs and CaTs at 0.5 Hz when introducing 0.925 mV left-shift in $Ca_V1.2$ activation, without (A) and with (B) CaMKII inhibition.

Supplementary Tables

Parameter	Initial Value	Fitted Value
mss p1	39.57	45.68
mss p2	9.87	6.36
hss p1	82.9	72.28
hss p2	6.09	5.95
tauj p1	2.04	0.09
tauj p2	0.02	0.02
tauj p3	100.60	93.64
tauj p4	8.28	9.52
tauj p5	0.31	0.79
tauj p6	0.99	0.88
tauj p7	38.45	97.10
tau hlate	200	175
GNa fast	75	48
GNa late	0.0075	0.006

Table S1. Fitting of wild-type Na^+ gating parameters

Parameter	Initial Value	Fitted Value
xs1ss p1	11.60	-13.86
xs1ss p2	8.93	23.71
GKs	0.0034	0.1303

Table S2. Fitting of wild-Type I_{Ks} gating parameters

Parameter	Initial Value	Fitted Value
xrss p1	48.0294	30.0
xrss p2	10.8758	7.0
tau xr p1	0.0018	0.00083
tau xr p2	4.74791	2.189
tau xr p3	0.1132	0.128035
tau xr p4	0.00061	0.00059
tau xr p5	35.2249	36.18555
tau xr p6	0.07	0.14506
rkr p1	12.0366	12.0
rkr p2	20.9951	16.0
GKr	0.024	0.013

Table S3. Fitting of wild-Type I_{Kr} gating parameters

Parameter	Initial Value	Fitted Value
ak1 p1	0.45	0.0001
aki p2	0.10	0.0897
aki p3	-28.28	-18.45
bki p1	1.48	0.0004
bki p2	0.0078	0.012
bki p3	8.7	5.63
bki p4	0.06	0.18
bki p5	-594.31	-1171.06
bki p6	-0.51	-0.1001
bki p7	4.75	2.35
GK1	0.95	1.15

Table S4. Fitting of wild-Type I_{K1} gating parameters

Parameter	Initial Value	Fitted Value
Km	0.1502	0.189818
aHill	2	1.756482
tau aa	1	0.845868
rm p1	-	4.999674
rm p2	-	10.420643
PCl	0.00014	0.0001668

Table S5. Fitting of wild-Type I_{to2} gating parameters

Parameter	Initial Value	Fitted Value
dss p1	3.94	7.370368
dss p2	4.23	4.52243
td p1	0.6	0.521699
td p2	0.05	0.035786
td p3	6	3.318584
td p4	0.09	0.097746
td p5	14	14.090054
jcass p1	19.58	23.707057
jcass p2	3.969	5.015585
tjca p1	35	41.883987
tjca p2	350	196.585546
tjca p3	20	8.741029
tjca p4	2	1.715899
tjca p5	100	85.19949
f1ss p1	0.8	0.671371
f1ss p2	19.58	26.220924
f1ss p3	3.696	5.756253
f1ss p4	0.2	0.42097
tf1 p1	70	13.525373
tf1 p2	1.2	0.947658
tf1 p3	0.0045	0.003223
tf1 p4	20	26.45832
tf1 p5	50	39.722335
tf1 p6	0.0045	0.0048
tf1 p7	30	26.721824
tf1 p8	10	10.117712
tf2 p1	100	121.300281
tf2 p2	-	-0.000796
tf2 p3	-	0.003154
tf2 p4	-	2.3316
tf2 p5	-	99.265781
tf2 p6	-	0.003924
tf2 p7	-	7.623763
tf2 p8	-	3.478524

Table S6. Fitting of wild-type I_{CaL} gating parameters (Vmode)

Parameter	Initial Value	Fitted Value
PCa	0.0009	0.00006
Kmn	0.05	0.0002
tau j	1	1.01
KCDI	9	24.33
Bt	4.75	2.73
Vmax SERCA	0.004375	0.002777
Kd SERCA	0.000092	0.00051
SR Leak	0.003938	0.002593

Table S7. Wild-Type, fitting of I_{CaL} gating parameters (Vmode+Cmode) and parameters affecting cellular Ca^{2+} fluxes.

Parameter	Initial Value	Optimized Value
PCa	0.00006	0.0000713
Bt	4.75	11.566485
tau jrel	0.0123	0.019455
Vmax SERCA	0.002777	0.002022
Kd SERCA	0.000509	0.000573
SR leak	0.002593	0.002926
GNa	48	45.0078240
GNa late	0.006	0.0077446
PCl	1.67E-04	0.0001859
GKr	0.013	0.0069036
GKs	0.13026	0.1441586
GK1	1.14962	0.5804041
GNaCa	0.0008	0.0004969
GNaK	30	44.3879700

Table S8. Parameter modifications after automatic optimization procedure.

References

1. Porta-Sánchez, A. *et al.* Unexpected impairment of INa underpins reentrant arrhythmias in a knock-in swine model of timothy syndrome. *Nat. Cardiovasc. Res.* **2**, 1291–1309 (2023).

2. Splawski, I. *et al.* Ca(V)1.2 calcium channel dysfunction causes a multisystem disorder including arrhythmia and autism. *Cell* **119**, 19–31 (2004).

3. Kumawat, A. *et al.* Molecular insights into the rescue mechanism of an hERG activator against severe LQT2 mutations (2024).

APPENDIX: Model Equations

1 Extracellular Ionic Concentrations

$[\mathrm{Na}]_o = 140.0$ mM (Extracellular Na^+ concentration)

$[\mathrm{Ca}]_o = 1.8$ mM (Extracellular Ca^{2+} concentration)

$[\mathrm{K}]_o = 4.0$ mM (Extracellular K^+ concentration)

$[\mathrm{Cl}]_o = 150.0$ mM (Extracellular Cl^- concentration)

2 Physical Constants

$R = 8314.0$ J mol^{-1} K^{-1} (Universal Gas constant)

$T = 310.0$ K (Temperature)

$F = 96485.0$ C/mol (Faraday's Constant)

3 Reversal Potentials

$$E_{\mathrm{Na}} = \frac{R \cdot T}{F} \cdot \ln\left(\frac{[Na]_o}{[Na]_i}\right)$$ (Reversal potential for Na^+)

$$E_{\mathrm{K}} = \frac{R \cdot T}{F} \cdot \ln\left(\frac{[K]_o}{[K]_i}\right)$$ (Reversal potential for K^+)

$\mathrm{PR}_{\mathrm{Na,K}} = 0.01833$ (Permeability ratio of Na^+ to K^+)

$$E_{\mathrm{Ks}} = \frac{R \cdot T}{F} \cdot \ln\left(\frac{[K]_o + \mathrm{PR}_{\mathrm{Na,K}} \cdot [Na]_o}{[K]_i + \mathrm{PR}_{\mathrm{Na,K}} \cdot [Na]_i}\right)$$ (Reversal potential for I_{Ks})

$$E_{\mathrm{Cl}} = \frac{R \cdot T}{F} \cdot \ln\left(\frac{[Cl]_i}{[Cl]_o}\right)$$ (Reversal potential for Cl^-)

4 Cell Geometry

$L = 0.0105$ cm (Cell length)

$\mathrm{r} = 0.0012$ cm (Cell radius)

$v_{\mathrm{cell}} = 1000 \cdot 3.14 \cdot \mathrm{rad}^2 \cdot L$ µL (Cell volume)

$A_{\mathrm{geo}} = 2 \cdot 3.14 \cdot \mathrm{rad}^2 + 2 \cdot 3.14 \cdot \mathrm{rad} \cdot L$ cm^2 (Geometric area)

$A_{\mathrm{cap}} = 2 \cdot A_{\mathrm{geo}}$ cm^2 (Capacitive area)

$v_{\mathrm{myo}} = 0.68 \cdot v_{\mathrm{cell}}$ (Volume of cytoplasm)

$v_{\mathrm{nsr}} = 0.0552 \cdot v_{\mathrm{cell}}$ (Volume of non-junctional SR)

$v_{\mathrm{jsr}} = 0.0048 \cdot v_{\mathrm{cell}}$ (Volume of junctional SR)

$v_{\mathrm{ss}} = 0.02 \cdot v_{\mathrm{cell}}$ (Volume of subspace)

5 Sodium Current (I_{Na}):

$$m_{\infty} = \frac{1.0}{1.0 + \exp\left(-\frac{V_m + 45.68}{6.36}\right)}$$

$$\tau_m = 0.3536 \cdot 2.8 \cdot \left(\frac{1.0}{6.765 \cdot \exp\left(\frac{V_m + 11.64}{34.77}\right) + 8.552 \cdot \exp\left(-\frac{V_m + 77.42}{5.955}\right)}\right)$$

$$\frac{dm}{dt} = \frac{m_{\infty} - m}{\tau_m}$$

$$h_{\infty} = \frac{1.0}{1 + \exp\left(\frac{V_m + 72.28}{5.95}\right)}$$

$$\tau_{hf} = 0.3536 \cdot 2.8 \left(\frac{1.0}{1.432 \cdot 10^{-5} \cdot \exp\left(-\frac{V_m + 1.196}{6.285}\right) + 6.149 \cdot \exp\left(\frac{V_m + 0.5096}{20.27}\right)}\right)$$

$$\tau_{hs} = 0.3536 \cdot 2.8 \left(\frac{1.0}{0.009794 \cdot \exp\left(-\frac{V_m + 17.95}{28.05}\right) + 0.3343 \cdot \exp\left(\frac{V_m + 5.730}{56.66}\right)}\right)$$

$$A_{hf} = 0.99$$

$$A_{hs} = 1.0 - A_{hf}$$

$$\frac{dh_f}{dt} = \frac{h_{\infty} - hf}{\tau_{hf}}$$

$$\frac{dh_s}{dt} = \frac{h_{\infty} - hs}{\tau_{hs}}$$

$$h = A_{hf} \cdot hf + A_{hs} \cdot hs$$

$$j_{\infty} = h_{\infty}$$

$$\tau_j = 2.8 \left(0.09 + \frac{1.0}{0.02 \cdot \exp\left(-\frac{V_m + 93.64}{9.52}\right) + 0.79 \cdot \exp\left(\frac{V_m + 0.88}{97.10}\right)}\right)$$

$$\frac{dj}{dt} = \frac{j_{\infty} - j}{\tau_j}$$

$$h_{CaMK,\infty} = \frac{1.0}{1 + \exp\left(\frac{V_m + 78.48}{5.95}\right)}$$

$$\tau_{hs,CaMK} = 3.0 \cdot \tau_{hs}$$

$$\frac{dh_{s,CaMK}}{dt} = \frac{h_{CaMK,\infty} - h_{s,CaMK}}{\tau_{hs,CaMK}}$$

$$h_{CaMK} = A_{hf} \cdot hf + A_{hs} \cdot h_{s,CaMK}$$

$$\tau_{j,CaMK} = 1.46 \cdot \tau_j$$

$$\frac{dj_{CaMK}}{dt} = \frac{j_{\infty} - j_{CaMK}}{\tau_{j,CaMK}}$$

$$\phi_{INa,CaMK} = \frac{1.0}{1.0 + \frac{K_{m,CaMK}}{CaMK_{active}}}$$

$$\overline{G_{Na,fast}} = 0.9377 \cdot (48.0 - \frac{14.0}{1.0 + \left(\frac{0.4}{CaMKa}\right)^6})\text{mS/uF}$$

$$I_{Na,fast} = \overline{G_{Na,fast}} \cdot (V_m - E_{Na}) \cdot m^3 \left((1.0 - \phi_{INa,CaMK}) \cdot h \cdot j + \phi_{INa,CaMK} \cdot h_{CaMK} \cdot j_{CaMK}\right)$$

$$\overline{G_{Na,late}} = 0.0077 \cdot \left(1 + 1.5 \cdot \frac{1}{1 + (0.1/\text{CaMKa})^6}\right) \text{mS/uF}$$

$$m_{L,\infty} = \frac{1}{1+\exp\left(-\frac{V_m+42.85}{5.264}\right)};$$

$$\tau_{mL} = \tau_m;$$

$$\frac{dm_L}{dt} = \frac{m_{L,\infty} - m_L}{\tau_{mL}};$$

$$h_{L,\infty} = \frac{1}{1+\exp\left(\frac{V_m+87.61}{7.488}\right)};$$

$$\tau_{hL} = 175;$$

$$\frac{dh_L}{dt} = \frac{h_{L,\infty} - hL}{\tau_{hL}};$$

$$h_{L,CaMK,\infty} = \frac{1}{1+\exp\left(\frac{V_m+93.81}{7.488}\right)};$$

$$\tau_{hL,CaMK} = 3.0 \cdot \tau_{hL};$$

$$dh_{L,CaMK} = \frac{h_{L,CaMK,\infty} - h_{L,CaMK}}{\tau_{hL,CaMK}};$$

$$f_{INaL,CaMK} = \frac{1}{1+\frac{K_{m,CaMK}}{CaMK_{active}}};$$

$$I_{Na,late} = \overline{G_{Na,late}} \cdot (V_m - E_{Na}) \cdot m_L \cdot ((1-\phi_{INaL,CaMK}) \cdot h_L + \phi_{INaL,CaMK} \cdot h_{L,CaMK});$$

6 Calcium-activated Chloride Current (I_{to2})

$$P_{Cl} = 1.114639 \cdot \left(1.668 \times 10^{-4} - 0.0000798 \cdot \left(\frac{\phi_{TS}}{0.22}\right)\right)$$

$$K_{m,Ito2} = 0.1887635$$

$$\overline{I_{to2}} = P_{Cl} \cdot \left(\frac{V_m \cdot F \cdot F}{R \cdot T}\right) \cdot \frac{[Cl]_i - [Cl]_o \cdot \exp\left(V_m \cdot \frac{F}{R \cdot T}\right)}{1-\exp\left(V_m \cdot \frac{F}{R \cdot T}\right)}$$

$$a_\infty = \frac{1.0}{1.0 + \left(\frac{K_{m,Ito2}}{[Ca]_{ss}}\right)^{1.746723}}$$

$$\tau_a = 0.857616$$

$$\frac{da}{dt} = \frac{a_\infty - a}{\tau_a}$$

$$TS_{Vshift} = 20.7 \cdot \frac{\phi_{TS}}{0.22}$$

$$r_{Ito2} = \frac{1.0}{1.0+\exp\left(-\frac{V_m-(4.99+TS_{Vshift})}{10.22}\right)}$$

$$I_{to2} = \overline{I_{to2}} \cdot a \cdot r_{Ito2}$$

7 L-Type Calcium Current (I_{CaL})

α and β rates (C $\longleftrightarrow$ O)

$$d_\infty = \frac{1.0}{1.0 + \exp\left(-\frac{V_m + 7.37}{4.52}\right)}$$

$$\tau_d = 0.52 + \frac{1.0}{\exp(-0.04 \cdot (V_m + 3.32)) + \exp(0.10 \cdot (V_m + 14.09))}$$

$$\alpha = \frac{d_\infty}{\tau_d}$$

$$\beta = \frac{1.0 - d_\infty}{\tau_d}$$

ψ and ω rates (I2 $\longleftrightarrow$ C)

$$f_\infty = \frac{1}{1 + \exp\left(\frac{V_m + 23.71}{5.02}\right)}$$

$$\tau_{fCa} = 41.88 + 196.59 \cdot \exp\left(-\frac{(V_m + 8.74)^2}{1.72 \cdot 85.20}\right)$$

$$\omega = \frac{f_\infty}{\tau_{fCa}}$$

$$\psi = \frac{1 - f_\infty}{\tau_{fCa}}$$

$$\psi_{TS} = 0.1 \cdot \psi \qquad \text{(For TS mutated channels)}$$

γ and δ rates (O $\longleftrightarrow$ I1)

$$f1_{\infty,0} = -0.09 + \left(\frac{0.67}{1.0 + \exp\left(\frac{V_m + 26.22}{5.76}\right)} + 0.42\right)$$

$$\tau_{f1,0} = 13.53 + \frac{0.95}{0.0032 \exp\left(\frac{V_m + 26.46}{-39.72}\right) + 0.0048 \exp\left(\frac{V_m + 26.72}{10.12}\right)}$$

$$k_{\tau,CaMK} = 2.5$$

$$\gamma_{VD} = \frac{1.0 - f1_{\infty_0}}{\tau_{f1,0}}$$

$$\delta_{VD} = \frac{f1_{\infty_0}}{\tau_{f1,0}}$$

$$\gamma_{VD,CaMK} = \frac{\gamma_{VD}}{k_{\tau,CaMK}}$$

$$\delta_{VD,CaMK} = \frac{\delta_{VD}}{k_{\tau,CaMK}}$$

$$\gamma_{CD} = \gamma_{VD} \cdot kCDI$$

$$\delta_{CD} = \delta_{VD} \cdot kCDI$$

$$\gamma_{CD,CaMK} = \gamma_{VD,CaMK} \cdot kCDI$$

$$\delta_{CD,CaMK} = \delta_{VD,CaMK} \cdot kCDI$$

$$\gamma_{VD,\,TS} = 0.06 \cdot \gamma_{VD} \qquad \text{(For TS mutated channels)}$$

$$\gamma_{VD,CaMK,\,TS} = \frac{\gamma_{VD,\,TS}}{k_{\tau,CaMK}} \qquad \text{(For TS mutated channels)}$$

$$\gamma_{CD,\,TS} = \gamma_{VD,\,TS} \cdot kCDI \qquad \text{(For TS mutated channels)}$$

$$\gamma_{CD,CaMK,\,TS} = \gamma_{VD,CaMK,\,TS} \cdot kCDI \qquad \text{(For TS mutated channels)}$$

θ and η rates (I1 $\longleftrightarrow$ I2)

$$\tau_{f2} = 1.0\left(121.30 + \frac{-0.0008}{0.0032\cdot exp(\frac{V_m+2.33}{-99.26}) + 0.0039\cdot exp(\frac{V_m+7.62}{3.48})}\right)$$

$$\tau_{f2,VD} = \tau_{f2}$$

$$\tau_{f2,CD} = \frac{\tau_{f2,VD}}{kCDI}$$

$$\tau_{f2,VD,CaMK} = \tau_{f2}\cdot k_{\tau,CaMK}$$

$$\tau_{f2,CD,CaMK} = \tau_{f2,CD}\cdot k_{\tau,CaMK}$$

$$\theta_{VD} = \frac{\alpha\cdot\gamma_{VD}\cdot\psi_{VD}}{\tau_{f2,VD}\cdot(\alpha\cdot\gamma_{VD}\cdot\psi_{VD} + \beta\cdot\delta_{VD}\cdot\omega_{VD})}$$

$$\theta_{CD} = \frac{\alpha\cdot\gamma_{CD}\cdot\psi_{CD}}{\tau_{f2,CD}\cdot(\alpha\cdot\gamma_{CD}\cdot\psi_{CD} + \beta\cdot\delta_{CD}\cdot\omega_{CD})}$$

$$\theta_{VD,CaMK} = \frac{\alpha\cdot\gamma_{VD,CaMK}\cdot\psi_{VD,CaMKI}}{\tau_{f2,VD,CaMK}\cdot(\alpha\cdot\gamma_{VD,CaMK}\cdot\psi_{VD,CaMK} + \beta\cdot\delta_{VD,CaMK}\cdot\omega_{VD,CaMK})}$$

$$\theta_{CD,CaMK} = \frac{\alpha\cdot\gamma_{CD,CaMK}\cdot\Psi_{CD,CaMK}}{\tau_{f2,CD,CaMK}\cdot(\alpha\cdot\gamma_{CD,CaMK}\cdot\psi_{CD,CaMK} + \beta\cdot\delta_{CD,CaMK}\cdot\omega_{CD,CaMK})}$$

$$\eta_{VD} = \frac{1.0}{\tau_{f2,VD}} - \theta_{VD}$$

$$\eta_{VD,CaMK} = \frac{1.0}{\tau_{f2,VD,CaMK}} - \theta_{VD,CaMK}$$

$$\eta_{CD} = \frac{1.0}{\tau_{f2,CD}} - \theta_{CD}$$

$$\eta_{CD,CaMK} = \frac{1.0}{\tau_{f2,CD,CaMK}} - \theta_{CD,CaMK}$$

Transitions between VDI and CDI loops

$$r_{\text{down}} = 0.1;$$

$$r_{\text{up}} = r_{\text{down}}\cdot\frac{\text{n}}{1-\text{n}};$$

$$\frac{dn}{dt} = \alpha_n\cdot k_{2,n} - \text{n}\cdot k_{-2,n}$$

$$\alpha_n = \frac{1-\text{n}}{(1+\frac{K_{m,n}}{[Ca]_{ss}})^4}$$

$$\frac{dj_n}{dt} = \frac{j_{n,\infty} - j_n}{\tau_{jn}}$$

$$j_{n,\infty} = \frac{1}{1+\exp\left(\frac{V_m+19.58+5}{3.696}\right)}$$

$$\tau_{jn} = 1.01$$

$$k_{-2,n} = j_n\cdot 150$$

$$K_{m,n} = 0.0002$$

$$k_{2,n} = 1000$$

$$kCDI = 24.33$$

Markov Model: VDI States

$$\frac{dO_{VD}}{dt} = \alpha \cdot C_{VD} + \delta_{VD} \cdot I1_{VD} - (\beta + \gamma_{VD}) \cdot O_{VD} - r_{\text{up}} \cdot O_{VD} + r_{\text{down}} \cdot O_{CD}$$
$$\frac{dI2_{VD}}{dt} = \eta_{VD} \cdot I1_{VD} + \psi_{VD} \cdot C_{VD} - (\theta_{VD} + \omega_{VD}) \cdot I2_{VD} - r_{\text{up}} \cdot I2_{VD} + r_{\text{down}} \cdot I2_{CD}$$
$$\frac{dI1_{VD}}{dt} = \theta_{VD} \cdot I2_{VD} + \gamma_{VD} \cdot O_{VD} - (\eta_{VD} + \delta_{VD}) \cdot I1_{VD} - r_{\text{up}} \cdot I1_{VD} + r_{\text{down}} \cdot I1_{CD}$$
$$\frac{dC_{VD}}{dt} = \beta \cdot O_{VD} + \omega_{VD} \cdot I2_{VD} - (\psi_{VD} + \alpha) \cdot C_{VD} - r_{\text{up}} \cdot C_{VD} + r_{\text{down}} \cdot C_{CD}$$

$$\begin{aligned}
\frac{dO_{VD,CaMK}}{dt} &= \alpha \cdot C_{VD,CaMK} + \delta_{VD,CaMK} \cdot I1_{VD,CaMK} - (\beta + \gamma_{VD,CaMK}) \cdot O_{VD,CaMK} \\
&\quad - r_{\text{up}} \cdot O_{VD,CaMK} + r_{\text{down}} \cdot O_{CD,CaMK}; \\
\frac{dI2_{VD,CaMK}}{dt} &= \eta_{VD,CaMK} \cdot I1_{VD,CaMK} + \psi_{VD,CaMK} \cdot C_{VD,CaMK} \\
&\quad - (\theta_{VD,CaMK} + \omega_{VD,CaMK}) \cdot I2_{VD,CaMK} - r_{\text{up}} \cdot I2_{VD,CaMK} \\
&\quad + r_{\text{down}} \cdot I2_{CD,CaMK}; \\
\frac{dI1_{VD,CaMK}}{dt} &= \theta_{VD,CaMK} \cdot I2_{VD,CaMK} + \gamma_{VD,CaMK} \cdot O_{VD,CaMK} \\
&\quad - (\eta_{VD,CaMK} + \delta_{VD,CaMK}) \cdot I1_{VD,CaMK} - r_{\text{up}} \cdot I1_{VD,CaMK} \\
&\quad + r_{\text{down}} \cdot I1_{CD,CaMK}; \\
\frac{dC_{VD,CaMK}}{dt} &= \beta \cdot O_{VD,CaMK} + \omega_{VD,CaMK} \cdot I2_{VD,CaMK} \\
&\quad - (\psi_{VD,CaMK} + \alpha) \cdot C_{VD,CaMK} - r_{\text{up}} \cdot C_{VD,CaMK} \\
&\quad + r_{\text{down}} \cdot C_{CD,CaMK};
\end{aligned}$$

Markov Model: CDI States

$$\frac{dI2_{CD}}{dt} = \eta_{CD} \cdot I1_{CD} + \psi_{CD} \cdot C_{CD} - (\theta_{CD} + \omega_{CD}) \cdot I2_{CD} + r_{\text{up}} \cdot I_{2_k} - r_{\text{down}} \cdot I2_{CD};$$
$$\frac{dI1_{CD}}{dt} = \theta_{CD} \cdot I2_{CD} + \gamma_{CD} \cdot O_{CD} - (\eta_{CD} + \delta_{CD}) \cdot I1_{CD} + r_{\text{up}} \cdot I_{1_k} - r_{\text{down}} \cdot I1_{CD};$$
$$\frac{dC_{CD}}{dt} = \beta \cdot O_{CD} + \omega_{CD} \cdot I2_{CD} - (\psi_{CD} + \alpha) \cdot C_{CD} + r_{\text{up}} \cdot C_k - r_{\text{down}} \cdot C_{CD};$$
$$O_{CD} = 1 - (C_{CD} + I1_{CD} + I2_{CD} + C_{VD} + I1_{VD} + I2_{VD} + O_{VD}).$$

$$\begin{aligned}
\frac{dI2_{CD,CaMK}}{dt} &= \eta_{CD,CaMK} \cdot I1_{CD,CaMK} + \psi_{CD,CaMK} \cdot C_{CD,CaMK} \\
&\quad - (\theta_{CD,CaMK} + \omega_{CD,CaMK}) \cdot I2_{CD,CaMK} + r_{\text{up}} \cdot I2_{VD,CaMK} \\
&\quad - r_{\text{down}} \cdot I2_{CD,CaMK}; \\
\frac{dI1_{CD,CaMK}}{dt} &= \theta_{CD,CaMK} \cdot I2_{CD,CaMK} + \gamma_{CD,CaMK} \cdot O_{CD,CaMK} \\
&\quad - (\eta_{CD,CaMK} + \delta_{CD,CaMK}) \cdot I1_{CD,CaMK} + r_{\text{up}} \cdot I1_{VD,CaMK} \\
&\quad - r_{\text{down}} \cdot I1_{CD,CaMK}; \\
\frac{dC_{CD,CaMK}}{dt} &= \beta \cdot O_{CD,CaMK} + \omega_{CD,CaMK} \cdot I2_{CD,CaMK} - (\psi_{CD,CaMK} + \alpha) \cdot C_{CD,CaMK} \\
&\quad + r_{\text{up}} \cdot C_{k,CaMK} - r_{\text{down}} \cdot C_{CD,CaMK}; \\
O_{CD,CaMK} &= 1 - (C_{CD,CaMK} + I1_{CD,CaMK} + I2_{CD,CaMK} + C_{VD,CaMK} \\
&\quad + I1_{VD,CaMK} + I2_{VD,CaMK} + O_{VD,CaMK})
\end{aligned}$$

Driving Force and Current Formulation

$$\Psi_{Ca} = z_{Ca}^2 \cdot \frac{V\mathrm{F}^2}{RT} \cdot \frac{(\gamma_{\mathrm{Ca}} \cdot [\mathrm{Ca}]_{\mathrm{ss}} \cdot \exp(\frac{z_{Ca}VF}{RT}) - \gamma_{\mathrm{Cao}} \cdot [\mathrm{Ca}]_{\mathrm{o}})}{\exp\left(\frac{z_{Ca}VF}{RT}\right) - 1}$$

$$z_{\mathrm{Ca}} = 2$$
$$\gamma_{\mathrm{Ca}} = 1.2$$
$$\gamma_{\mathrm{Cao}} = 0.341$$
$$P_{\mathrm{Ca}} = 0.0000713$$
$$P_{\mathrm{Ca},CaMK} = 1.1 \times P_{\mathrm{Ca}}$$
$$\phi I_{\mathrm{CaL,CaMK}} = \frac{1}{1 + \frac{K_{mCaMK}}{CaMK_{\mathrm{active}}}} \quad \text{(Fraction of channels phosphorylated by CaMK)}$$
$$I_{\mathrm{CaL,total}} = (P_{\mathrm{Ca,CaMK}} \cdot O_{\mathrm{VD,CaMK}} \cdot \Psi_{\mathrm{Ca}} + P_{\mathrm{Ca,CaMK}} \cdot O_{\mathrm{CD,CaMK}} \cdot \Psi_{\mathrm{Ca}}) \cdot \phi I_{\mathrm{CaL,CaMK}} + (P_{\mathrm{Ca}} \cdot O_{\mathrm{VD}} \cdot \Psi_{\mathrm{Ca}} + P_{\mathrm{Ca}} \cdot O_{\mathrm{CD}} \cdot \Psi_{\mathrm{Ca}}) \cdot (1 - \phi I_{\mathrm{CaL,CaMK}})$$

8 Rapid Delayed Rectifier K$^+$ Current (I_{Kr})

$$x_{r_\infty} = \frac{1}{1 + \exp\left(-\frac{V_m+30}{7}\right)}$$
$$\tau_{xr} = \frac{1}{\left(\frac{0.00083\cdot(V_m+2.189)}{1-\exp(-0.13\cdot(V_m+2.189))}\right) + \left(\frac{0.00059\cdot(V_m+36.19)}{\exp(0.145\cdot(V_m+36.19))-1.0}\right)}$$
$$\frac{dx_r}{dt} = \frac{x_{r_\infty} - x_r}{\tau_{xr}}$$
$$r_{Kr} = \frac{1}{1 + \exp\left(\frac{V_m+12.0}{16.0}\right)}$$
$$\overline{G}_{Kr} = 0.007$$
$$I_{Kr} = \overline{G}_{Kr}\sqrt{\frac{[K]_o}{5.4}} \cdot x_r \cdot r_{Kr} \cdot (V_m - E_K)$$

9 Slow Delayed Rectifier K$^+$ Current (I_{Ks})

$$x_{s1,\infty} = \frac{1}{1+\exp\left(-\frac{V_m-13.86}{23.71}\right)}$$

$$\tau_{xs1} = 817.3 + \frac{1}{2.33\times 10^{-4}\exp\left(\frac{V_m+48.28}{17.8}\right)+0.001292\exp\left(-\frac{V_m+210}{230}\right)}$$

$$\frac{dx_{s1}}{dt} = \frac{x_{s1,\infty}-x_{s1}}{\tau_{xs1}}$$

$$x_{s2,\infty} = x_{s1,\infty}$$

$$\tau_{xs2} = \frac{1}{0.01\exp\left(\frac{V_m-50.0}{20.0}\right)+0.0193\exp\left(-\frac{V_m+66.54}{31.0}\right)}$$

$$\frac{dx_{s2}}{dt} = \frac{x_{s2,\infty}-x_{s2}}{\tau_{xs2}}$$

$$Ks_{Ca} = 1.0 + \frac{0.6}{1.0+\left(\frac{3.8\times 10^{-5}}{[\mathrm{Ca}]_i}\right)^{1.4}}$$

$$\overline{G_{Ks}} = 0.144\cdot\left(1-0.4\cdot\frac{\phi_{TS}}{0.22}\right)$$

$$I_{Ks} = \overline{G_{Ks}}\cdot Ks_{Ca}\cdot x_{s1}\cdot x_{s2}\cdot(V_m-E_{Ks})$$

10 Inward Rectifier K$^+$ Current (I_{K1})

$$\alpha_{K1} = \frac{0.000131}{1+\exp\left(0.09\cdot(V_m-E_K-18.45)\right)}$$

$$\beta_{K1} = \frac{0.0004\cdot\exp\left(0.0119\cdot(V_m-E_K+5.62)\right)+\exp\left(0.18\cdot(V_m-E_K-1171.06)\right)}{1+\exp\left(-0.10\cdot(V_m-E_K+2.37)\right)}$$

$$R_{K1} = \frac{\alpha_{K1}}{\alpha_{K1}+\beta_{K1}}$$

$$\overline{G_{K1}} = 0.58\cdot\sqrt{\frac{[K]o}{5.4}}$$

$$I_{K1} = \overline{G_{K1}}\cdot R_{K1}\cdot(V_m-E_K)$$

11 Na$^+$/Ca^{2+} Exchange Current (I_{NaCa})

For $\gamma \in \{\mathrm{i,ss}\}$

$k_{Na1} = 15.0\,\mathrm{mM}$

$k_{Na2} = 5.0\,\mathrm{mM}$

$k_{Na3} = 88.12\,\mathrm{mM}$

$k_{asymm} = 12.5$

$\omega_{Na} = 6.0\times 10^4\,\mathrm{Hz}$

$\omega_{Ca} = 6.0\times 10^4\,\mathrm{Hz}$

$\omega_{Na,Ca} = 5.0\times 10^3\,\mathrm{Hz}$

$k_{Ca,on} = 1.5\times 10^6\,\mathrm{mM/ms}$

$k_{Ca,off} = 5.0\times 10^3\,\mathrm{Hz}$

$$q_{Na} = 0.5224$$
$$q_{Ca} = 0.1670$$
$$h_{Ca} = \exp\left(\frac{q_{Ca} V_m F}{RT}\right)$$
$$h_{Na} = \exp\left(\frac{q_{Na} V_m F}{RT}\right)$$
$$h_1 = 1 + \frac{[Na]_\gamma}{k_{Na3}}(1 + h_{Na})$$
$$h_2 = \frac{[Na]_\gamma \cdot h_{Na}}{k_{Na3} \cdot h1}$$
$$h_3 = \frac{1.0}{h_1}$$
$$h_4 = 1.0 + \frac{[Na]_\gamma}{k_{Na1}}\left(1.0 + \frac{[Na]_\gamma}{k_{Na2}}\right)$$
$$h_5 = \frac{{[Na]_\gamma}^2}{h4 \cdot k_{Na1} \cdot k_{Na2}}$$
$$h_6 = \frac{1.0}{h_4}$$
$$h_7 = 1.0 + \frac{[Na]_o}{k_{Na3}}\left(1.0 + \frac{1.0}{h_{Na}}\right)$$
$$h_8 = \frac{[Na]_o}{k_{Na3} \cdot h_{na} \cdot h7}$$
$$h_9 = \frac{1.0}{h_7}$$
$$h_{10} = k_{asymm} + 1.0 + \frac{[Na]_o}{k_{Na1}}\left(1.0 + \frac{[Na]_o}{k_{Na2}}\right)$$
$$h_{11} = \frac{{[Na]_o}^2}{h_1 0 \cdot k_{Na1} \cdot k_{Na2}}$$
$$h_{12} = \frac{1.0}{h_{10}}$$
$$k_1 = h_{12} \cdot [Ca]_o \cdot k_{Ca,on}$$
$$k_2 = k_{Ca,off}$$
$$k_3' = h_9 \cdot \omega_{Ca}$$
$$k_3'' = h_8 \cdot \omega_{NaCa}$$
$$k_3 = k_3' + k_3''$$
$$k_4' = \frac{h_3 \cdot \omega_{Ca}}{h_{Ca}}$$
$$k_4'' = h_2 \omega_{NaCa}$$
$$k_4 = k_4' + k_4''$$
$$k_5 = k_{Ca,off}$$
$$k_6 = h_6 \cdot [Ca]_\gamma \cdot k_{Ca,on}$$
$$k_7 = h_5 \cdot h_2 \cdot \omega_{Na}$$
$$k_8 = h_8 \cdot h_1 1 \cdot \omega_{Na}$$
$$x_1 = k_2 \cdot k_4 \cdot (k_7 + k_6) + k_5 \cdot k_7 \cdot (k_2 + k_3)$$
$$x_2 = k_1 \cdot k_7 \cdot (k_4 + k_5) + k_4 \cdot k_6 \cdot (k_1 + k_8)$$
$$x_3 = k_1 \cdot k_3 \cdot (k_7 + k_6) + k_8 \cdot k_6 \cdot (k_2 + k_3)$$
$$x_4 = k_2 \cdot k_8 \cdot (k_4 + k_5) + k_3 \cdot k_5 \cdot (k_1 + k_8)$$

$$E_1 = \frac{x_1}{x_1 + x_2 + x_3 + x_4}$$
$$E_2 = \frac{x_2}{x_1 + x_2 + x_3 + x_4}$$
$$E_3 = \frac{x_3}{x_1 + x_2 + x_3 + x_4}$$
$$E_4 = \frac{x_4}{x_1 + x_2 + x_3 + x_4}$$
$$K_{mCaAct} = 150.0 \times 10^{-6}$$
$$allo_\gamma = \frac{1.0}{1.0 + \left(\frac{KmCaAct}{[Ca]_\gamma}\right)^2}$$
$$z_{Na} = 1.0$$
$$J_{NaCa,Na,\gamma} = 3.0 \cdot (E_4 \cdot k_7 - E_1 \cdot k_8) + E3 \cdot k4'' - E_2 \cdot k3''$$
$$J_{NaCa,Ca,\gamma} = E_2 \cdot k_2 - E_1 \cdot k_1$$
$$\overline{G_{NaCa}} = 0.0005 \mu A/\mu F$$
$$I_{NaCa,i} = \overline{G_{NaCa}} \cdot 0.8 \cdot allo_i \cdot (z_{Na} \cdot J_{NaCa,Na,i} + z_{Ca} \cdot J_{NaCa,Ca,i})$$
$$I_{NaCa,ss} = \overline{G_{NaCa}} \cdot 0.2 \cdot allo_{ss} \cdot (z_{Na} \cdot J_{NaCa,Na,ss} + z_{Ca} \cdot J_{NaCa,Ca,ss})$$
$$I_{NaCa} = I_{NaCa,i} + I_{NaCa,ss}$$

12 Na^+/K^+ ATPase Current (I_{NaK})

$$P_{NaK} = 44.39 \mu A/\mu F$$
$$k1^+ = 949.5$$
$$k1^- = 182.4$$
$$k2^+ = 687.2$$
$$k2^- = 39.4$$
$$k3^+ = 1899.0$$
$$k3^- = 79300.0$$
$$k4^+ = 639.0$$
$$k4^- = 40.0$$
$$K^o_{Nai} = 9.073$$
$$K^o_{Nao} = 27.78$$
$$\Delta = -0.1550$$
$$K_{Nai} = K^o_{Nai} \cdot exp\left(\frac{\Delta \cdot V_m \cdot F}{3.0 \cdot R \cdot T}\right)$$
$$K_{Nao} = K^o_{Nao} \cdot exp\left(\frac{(1-\Delta) \cdot V_m \cdot F}{3.0 \cdot R \cdot T}\right)$$
$$K_{Ki} = 0.5\,\text{mM}$$
$$K_{Ko} = 0.3582\,\text{mM}$$
$$[MgADP] = 0.05$$
$$[MgATP] = 4.0$$
$$K_{MgATP} = 1.698 \times 10^{-7}\,\text{mM}$$
$$[H^+] = 1.0 \times 10^{-7}\,\text{mM}$$

$$[\Sigma P] = 4.2\,\text{mM}$$
$$K_{HP} = 1.698 \times 10^{-7}\,\text{mM}$$
$$K_{Na,P} = 224.0\,\text{mM}$$
$$K_{K,P} = 292.0\,\text{mM}$$
$$[P] = [\Sigma P] / \left(1.0 + \frac{H}{K_{H,P}} + \frac{[Na]_i}{K_{Na,P} + \frac{[K]_i}{K_{K,P}}} \right)$$
$$\alpha_1 = \frac{k_1^+ \left(\frac{[Na]_i}{K_{Nai}}\right)^3}{\left(1.0 + \frac{[Na]_i}{K_{Nai}}\right)^3 + \left(1 + \frac{[K]_i}{K_{Ki}}\right)^2 - 1}$$
$$\beta_1 = k_1^- \cdot [MgADP]$$
$$\alpha_2 = k_2^+$$
$$\beta_2 = \frac{k_2^- \left(\frac{[Na]_o}{K_{Nao}})^3\right)}{\left((1.0 + \frac{[Na]_o}{K_{Nao}}\right)^3 + \left(1.0 + \frac{[K]_o}{K_{Ko}}\right)^2 - 1}$$
$$\alpha_3 = \frac{k_3^+ \left(\frac{[K]_o}{K_{Ko}})^2\right)}{\left((1.0 + \frac{[Na]_o}{K_{Nao}}\right)^3 + \left(1.0 + \frac{[K]o}{K_{Ko}}\right)^2 - 1}$$
$$\beta_3 = \frac{k_3^- \cdot [P] \cdot [H^+]}{1 + \frac{[MgATP]}{K_{MgATP}}}$$
$$\alpha_4 = \frac{k_4^+ \cdot \frac{[MgATP]}{K_{MgATP}}}{1 + \frac{[MgATP]}{K_{MgATP}}}$$
$$\beta_4 = \frac{k_4^- \left(\frac{[K]_i}{K_{Ki}})^2\right)}{\left((1 + \frac{[Na]_i}{K_{Nai}}\right)^3 + \left(1 + \frac{[K]i}{K_{Ki}}\right)^2 - 1}$$
$$x_1 = \alpha_4 \cdot \alpha_1 \cdot \alpha_2 + \beta_2 \cdot \beta_4 \cdot \beta_3 + \alpha_2 \cdot \beta_4 \cdot \beta_3 + \beta_3 \cdot \alpha_1 \cdot \alpha_2$$
$$x_2 = \beta_2 \cdot \beta_1 \cdot \beta_4 + \alpha_1 \cdot \alpha_2 \cdot \alpha_3 + \alpha_3 \cdot \beta_1 \cdot \beta_4 + \alpha_2 \cdot \alpha_3 \cdot \beta_4$$
$$x_3 = \alpha_2 \cdot \alpha_3 \cdot \alpha_4 + \beta_3 \cdot \beta_2 \cdot \beta_1 + \beta_2 \cdot \beta_1 \cdot \alpha_4 + \alpha_3 \cdot \alpha_4 \cdot \beta_1$$
$$x_4 = \beta_4 \cdot \beta_3 \cdot \beta_2 + \alpha_3 \cdot \alpha_4 \cdot \alpha_1 + \beta_2 \cdot \alpha_4 \cdot \alpha_1 + \beta_3 \cdot \beta_2 \cdot \alpha_1$$
$$E_1 = x1/(x_1 + x_2 + x_3 + x_4)$$
$$E_2 = x2/(x_1 + x_2 + x_3 + x_4)$$
$$E_3 = x3/(x_1 + x_2 + x_3 + x_4)$$
$$E_4 = x4/(x_1 + x_2 + x_3 + x_4)$$
$$z_K = 1.0$$
$$J_{NaK,Na} = 3 \cdot (E_1 \cdot \alpha_3 - E_2 \cdot \beta_3)$$
$$J_{NaK,K} = 2 \cdot (E_4 \cdot \beta_1 - E_3 \cdot \alpha_1)$$
$$I_{Na,K} = P_{NaK} \cdot (z_{Na} \cdot J_{NaK,Na} + z_K \cdot J_{NaK,K})$$

13 Background Currents (I_{Nab}, I_{Cab}, I_{Kb}) and Sarcolemmal Ca^{2+} Pump Current (I_{pCa})

$$\overline{G_{Kb}} = 0.003\,\text{mS/uF}$$
$$x_{Kb} = \frac{1}{1+\exp\left(-\frac{V_m-14.48}{18.34}\right)}$$
$$I_{Kb} = \overline{G_{Kb}} \cdot x_{Kb} \cdot (V_m - E_K)$$
$$\overline{G_{Nab}} = 3.75 \times 10^{-10}\,\text{mS/uF}$$
$$I_{Nab} = \overline{G_{Nab}} \cdot {z_{Na}}^2 \cdot \frac{V_m \cdot F^2}{RT} \cdot \frac{[Na]_i \cdot exp\left(\frac{z_{Na}\cdot V\cdot F}{R\cdot T}\right) - [Na]_o}{\exp\left(\frac{z_{Na}^2\cdot V\cdot F}{RT}\right) - 1.0}$$

$$\overline{G_{Cab}} = 2.5 \times 10^{-8}\,\text{mS/uF}$$
$$I_{Cab} = \overline{G_{Cab}} \cdot {z_{Ca}}^2 \cdot \frac{V_m \cdot F^2}{RT} \cdot \frac{\gamma_{Cai} \cdot [Ca]_i \cdot exp\left(\frac{z_{Ca}\cdot V\cdot F}{R\cdot T}\right) - \gamma_{Cao} \cdot [Ca]_o}{\exp\left(\frac{z_{Ca}^2\cdot V\cdot F}{RT}\right) - 1.0}$$

$$\overline{G_{pCa}} = 0.0005\,\text{mS/uF}$$
$$I_{pCa} = \overline{G_{pCa}} \cdot \frac{[Ca]_i}{0.0005 + [Ca]_i}$$

14 Voltage

$$\frac{dV_m}{dt} = -(I_{Na,\text{fast}} + I_{Na,\text{late}} + I_{to2} + I_{CaL} + I_{CaNa} + I_{CaK} + I_{Kr} + I_{Ks} + I_{K1} + I_{NaCa,i} + I_{NaCa,ss} + I_{NaK} + I_{Nab} + I_{Kb} + I_{pCa} + I_{Cab} + I_{stim})$$

15 Ca^{2+}/Calmodulin-Dependent Protein Kinase (CaMK)

$$\alpha_{\text{CaMK}} = 0.05\,\text{ms}^{-1}$$
$$\beta_{\text{CaMK}} = 0.00068\,\text{ms}^{-1}$$
$$\text{CaMK}_0 = 0.05$$
$$\text{K}_{\text{mCaM}} = 0.0015\,\text{mM}$$

$$\text{CaMK}_{\text{bound}} = \text{CaMK}_0 \cdot \frac{1.0 - \text{CaMK}_{\text{trap}}}{1.0 + \frac{\text{K}_{\text{mCaM}}}{[Ca]_{ss}}}$$
$$\text{CaMK}_{\text{active}} = \text{CaMK}_{\text{bound}} + \text{CaMK}_{\text{trap}}$$
$$\frac{d\text{CaMK}_{\text{trap}}}{dt} = \alpha_{\text{CaMK}} \cdot \text{CaMK}_{\text{bound}} \cdot (\text{CaMK}_{\text{bound}} + \text{CaMK}_{\text{trap}}) - \beta_{\text{CaMK}} \cdot \text{CaMK}_{\text{trap}}$$

16 Diffusion Fluxes

$$\text{J}_{\text{diff,Na}} = \frac{[\text{Na}]_{ss} - [\text{Na}]_i}{2}$$
$$\text{J}_{\text{diff,Ca}} = \frac{[\text{Ca}]_{ss} - [\text{Ca}]_i}{2}$$
$$\text{J}_{\text{diff,K}} = \frac{[\text{K}]_{ss} - [\text{K}]_i}{2}$$

17 Ca^{2+} Fluxes

17.1 SR Ca^{2+} Release Flux, via RyR2 (J_{rel})

$$\beta_\tau = 11.57\,\text{ms}$$
$$\alpha_{\text{rel}} = 0.5 \cdot \beta_\tau$$
$$\tau_{\text{rel,NP}} = \frac{\beta_\tau}{1.0 + \left(\frac{0.0195}{[\text{Ca}]_{\text{JSR}}}\right)}, \tau_{\text{rel,NP}} \geq 0.001$$
$$\text{J}_{\text{rel,NP},\infty} = \frac{\alpha_{\text{rel}} \cdot (-I_{CaL})}{1.0 + \left(\frac{1.5}{[\text{Ca}]_{\text{JSR}}}\right)^8}$$
$$\frac{d\text{J}_{\text{rel,NP}}}{dt} = \frac{\text{J}_{\text{rel,NP},\infty} - \text{J}_{\text{rel,NP}}}{\tau_{\text{rel,NP}}}$$
$$\beta_{\tau,\text{CaMK}} = 1.25 \cdot \beta_\tau$$

$$\alpha_{\text{rel,CaMK}} = 0.5 \cdot \beta_{\tau,\text{CaMK}}$$
$$\tau_{\text{rel,CaMK}} = \frac{\beta_{\tau,\text{CaMK}}}{1.0 + \left(\frac{0.0004}{[\text{Ca}]_{\text{JSR}}}\right)}, \tau_{\text{rel,CaMK}} \geq 0.001$$
$$\text{J}_{\text{rel,CaMK},\infty} = \frac{\alpha_{\text{rel,CaMK}} \cdot (-I_{CaL})}{1.0 + \left(\frac{1.5}{[\text{Ca}]_{\text{JSR}}}\right)^8}$$
$$\frac{d\text{J}_{\text{rel,CaMK}}}{dt} = \frac{\text{J}_{\text{rel,CaMK},\infty} - \text{J}_{\text{rel,CaMK}}}{\tau_{\text{rel,CaMK}}}$$
$$\text{K}_{\text{m,CaMKII}} = 0.15$$
$$\phi_{\text{rel,CaMK}} = \frac{1}{1 + \frac{\text{K}_{\text{m,CaMKII}}}{\text{CamK}_{\text{active}}}}$$
$$J_{\text{rel}} = (1.0 - \phi_{\text{rel,CaMK}}) \cdot \text{J}_{\text{rel,NP}} + \phi_{\text{rel,CaMK}} \cdot \text{J}_{\text{rel,CaMK}}$$

17.2 Ca^{2+} Uptake Flux via SERCA Pump (J_{up})

$$\text{J}_{\text{up,NP}} = \frac{0.002022 \cdot [\text{Ca}]_i}{0.000573 + [\text{Ca}]_i}$$
$$\overline{\Delta\text{K}_{\text{m,PLB}}} = 0.00017\,\text{mM}$$
$$\overline{\Delta\text{J}_{\text{up,CaMK}}} = 1.75$$
$$\text{J}_{\text{up,CaMK}} = (1 + \overline{\Delta\text{J}_{\text{up,CaMK}}}) \cdot \frac{0.002022 \cdot [\text{Ca}]_i}{0.000573 - \overline{\Delta\text{K}_{\text{m,PLB}}} + [\text{Ca}]_i}$$
$$\text{J}_{\text{leak}} = \frac{0.002926 \cdot [\text{Ca}]_{\text{NSR}}}{15}$$
$$\text{K}_{\text{m,CaMKII}} = 0.15$$
$$\phi_{\text{up,CaMK}} = \frac{1}{1 + \frac{\text{K}_{\text{m,CaMKII}}}{\text{CamK}_{\text{active}}}}$$
$$J_{\text{up}} = \left(1.0 - \phi_{\text{up,CaMK}}\right) \cdot \text{J}_{\text{up,NP}} + \phi_{\text{up,CaMK}} \cdot \text{J}_{\text{up,CaMK}} - \text{J}_{\text{leak}}$$

17.3 Ca^{2+} Translocation from NSR to JSR (J_{tr})

$$\text{J}_{\text{tr}} = \frac{[\text{Ca}]_{\text{NSR}} - [\text{Ca}]_{\text{JSR}}}{100}$$

18 Buffers

$$\overline{\text{CMDN}} = 0.05\,\text{mM}$$

$$\text{K}_{\text{m,CMDN}} = 0.00238\,\text{mM}$$

$$\overline{\text{TRPN}} = 0.07\,\text{mM}$$

$$\text{K}_{\text{m,TRPN}} = 0.003388442\,\text{mM}$$

$$\overline{\text{BSR}} = 0.047\,\text{mM}$$

$$\text{K}_{\text{m,BSR}} = 0.00087\,\text{mM}$$

$$\overline{\text{BSL}} = 1.124\,\text{mM}$$

$$\text{K}_{\text{m,BSL}} = 0.0087\,\text{mM}$$

$$\overline{\text{CSQN}} = 10.0\,\text{mM}$$

$$\text{K}_{\text{m,CSQN}} = 0.8\,\text{mM}$$

$$\frac{d[\text{Na}]_i}{dt} = -\left(I_{Na} + I_{NaL} + 3\cdot I_{NaCa_i} + 3\cdot I_{NaK} + I_{Nab}\right)\cdot\frac{A_{\text{cap}}}{F\cdot \text{v}_{\text{myo}}} + J_{\text{diff,Na}}\cdot\frac{\text{v}_{\text{ss}}}{\text{v}_{\text{myo}}}$$

$$\frac{d[\text{Na}]_{\text{ss}}}{dt} = -\left(I_{CaNa} + 3\cdot I_{NaCa,ss}\right)\cdot\frac{A_{\text{cap}}}{F\cdot \text{v}_{\text{ss}}} - J_{\text{diff,Na}}$$

$$\frac{d[\text{K}]_i}{dt} = -\left(I_{to2} + I_{Kr} + I_{Ks} + I_{K1} + I_{\text{stim}} - 2\cdot I_{NaK}\right)\cdot\frac{A_{\text{cap}}}{F\cdot \text{v}_{\text{myo}}} + J_{\text{diff,K}}\cdot\frac{\text{v}_{\text{ss}}}{\text{v}_{\text{myo}}}$$

$$\frac{d[\text{K}]_{\text{ss}}}{dt} = -I_{CaK}\cdot\frac{A_{\text{cap}}}{F\cdot \text{v}_{\text{ss}}} - J_{\text{diff,K}}$$

$$\beta_{\text{Cai}} = \frac{1.0}{1.0 + \frac{\overline{\text{CMDN}}\cdot \text{K}_{\text{m,CMDN}}}{(\text{K}_{\text{m,CMDN}} + [\text{Ca}]_i)^2} + \frac{\overline{\text{TRPN}}\cdot \text{K}_{\text{m,TRPN}}}{(\text{K}_{\text{m,TRPN}} + [\text{Ca}]_i)^2}}$$

$$\frac{d[\text{Ca}]_i}{dt} = \beta_{\text{Cai}}\left(-\left(I_{pCa} + I_{Cab} - 2\cdot I_{NaCa_i}\right)\cdot\frac{A_{\text{cap}}}{2\cdot F\cdot \text{v}_{\text{myo}}} - \frac{J_{\text{up}}\cdot V_{nsr}}{\text{v}_{\text{myo}}} + \frac{J_{\text{diff,Ca}}\cdot V_{ss}}{\text{v}_{\text{myo}}}\right)$$

$$\beta_{Cass} = \frac{1.0}{1.0 + \frac{\overline{\text{BSR}}\cdot \text{K}_{\text{m,BSR}}}{(\text{K}_{\text{m,BSR}} + [\text{Ca}]_{ss})^2} + \frac{\overline{\text{BSL}}\cdot \text{K}_{\text{m,BSL}}}{(\text{K}_{\text{m,BSL}} + [\text{Ca}]_{ss})^2}}$$

$$\frac{d[\text{Ca}]_{ss}}{dt} = \beta_{\text{Cass}}\left(-\left(I_{CaL} + I_{Cab} - 2\cdot I_{NaCa_ss}\right)\cdot\frac{A_{\text{cap}}}{2\cdot F\cdot \text{v}_{\text{ss}}} + \frac{J_{\text{rel}}\cdot V_{jsr}}{\text{v}_{\text{ss}}} - J_{\text{diff,Ca}}\right)$$

$$\frac{d[\text{Ca}]_{nsr}}{dt} = J_{\text{up}} - J_{\text{tr}}\cdot\frac{\text{v}_{\text{jsr}}}{\text{v}_{\text{nsr}}}$$

$$\beta_{Cajsr} = \frac{1.0}{1.0 + \frac{\overline{\text{CSQN}}\cdot \text{K}_{\text{m,CSQN}}}{(\text{K}_{\text{m,CSQN}} + [\text{Ca}]_{jsr})^2}}$$

$$\frac{d[\text{Ca}]_{jsr}}{dt} = \beta_{Cajsr}\cdot\left(J_{\text{tr}} - J_{\text{rel}}\right)$$
